# Limited effects of long-term daily cranberry consumption on the gut microbiome in a placebo-controlled study of women with recurrent urinary tract infections

**DOI:** 10.1186/s12866-021-02106-4

**Published:** 2021-02-18

**Authors:** Timothy J. Straub, Wen-Chi Chou, Abigail L. Manson, Henry L. Schreiber, Bruce J. Walker, Christopher A. Desjardins, Sinéad B. Chapman, Kerrie L. Kaspar, Orsalem J. Kahsai, Elizabeth Traylor, Karen W. Dodson, Meredith A. J. Hullar, Scott J. Hultgren, Christina Khoo, Ashlee M. Earl

**Affiliations:** 1 Infectious Disease & Microbiome Program, Broad Institute, 415 Main Street, Cambridge, MA 02142 USA; 2 Department of Immunology and Infectious Diseases, Harvard T.H. Chan School of Public Health, Boston, MA USA; 3 Department of Molecular Microbiology, Washington University School of Medicine in St. Louis, St. Louis, MO USA; 4 Center for Women’s Infectious Disease Research, Washington University School of Medicine in St. Louis, St. Louis, MO USA; 5 Division of Biology & Biological Engineering, California Institute of Technology, Pasadena, CA USA; 6 Ocean Spray Cranberries, Lakeville-Middleboro, MA USA; 7 Public Health Sciences Division, Fred Hutchinson Cancer Research Center, Seattle, WA USA

**Keywords:** Urinary tract infection (UTI), Microbiome, Metagenome, *Flavonifractor*, Cranberry

## Abstract

**Background:**

Urinary tract infections (UTIs) affect 15 million women each year in the United States, with > 20% experiencing frequent recurrent UTIs. A recent placebo-controlled clinical trial found a 39% reduction in UTI symptoms among recurrent UTI sufferers who consumed a daily cranberry beverage for 24 weeks. Using metagenomic sequencing of stool from a subset of these trial participants, we assessed the impact of cranberry consumption on the gut microbiota, a reservoir for UTI-causing pathogens such as *Escherichia coli*, which causes > 80% of UTIs.

**Results:**

The overall taxonomic composition, community diversity, carriage of functional pathways and gene families, and relative abundances of the vast majority of observed bacterial taxa, including *E. coli*, were not changed significantly by cranberry consumption. However, one unnamed *Flavonifractor* species (OTU41), which represented ≤1% of the overall metagenome, was significantly less abundant in cranberry consumers compared to placebo at trial completion. Given *Flavonifractor’s* association with negative human health effects, we sought to determine OTU41 characteristic genes that may explain its differential abundance and/or relationship to key host functions. Using comparative genomic and metagenomic techniques, we identified genes in OTU41 related to transport and metabolism of various compounds, including tryptophan and cobalamin, which have been shown to play roles in host-microbe interactions.

**Conclusion:**

While our results indicated that cranberry juice consumption had little impact on global measures of the microbiome, we found one unnamed *Flavonifractor* species differed significantly between study arms. This suggests further studies are needed to assess the role of cranberry consumption and *Flavonifractor* in health and wellbeing in the context of recurrent UTI.

**Trial registration:**

Clinical trial registration number: ClinicalTrials.govNCT01776021.

**Supplementary Information:**

The online version contains supplementary material available at 10.1186/s12866-021-02106-4.

## Background

Urinary tract infections (UTIs) are among the most common bacterial infections, affecting 15 million women each year in the United States [1] and 150 million worldwide [2]. The economic burden of UTIs is over $3.5 billion per year in the United States alone [3]. UTIs are also often recurrent, with 20–30% of women experiencing recurrent UTIs (rUTIs), even after appropriate antibiotic treatment [4, 5]. Since UTI treatment accounts for over 15% of all antibiotics prescribed in the U.S., UTIs contribute significantly to the total burden of drug resistance [6]. Uropathogenic *E. coli* (UPEC) is responsible for more than 80% of UTIs and has been the focus of many research studies [7, 8]. A better understanding of how to control rUTIs and curb UPEC colonization and infection without repeated use of antibiotics could improve patients’ lives and help slow the propagation of antibiotic resistance [9–11].

One proposed approach to combating rUTI is prophylactic consumption of cranberry products; however, the efficacy of cranberry products in preventing UTI is not clear [12–24]. No specific in vivo mechanism has been determined to explain cranberry’s potential preventative effects, and possible mechanisms have only been described in vitro [25–30]. Several studies have proposed that components of cranberry may influence the micro-environment in the bladder directly [23, 24, 31, 32]. Other studies have suggested that cranberry may affect the microbial inhabitants in the gut, in addition to modulating levels of gastrointestinal inflammation [33–37]. Thus, in vivo evidence is needed to address this gap in knowledge.

To better understand the impacts of daily cranberry consumption on the gut microbiota, we examined the gut microbiome of women with a history of recent rUTI who consumed either cranberry or placebo beverage daily for 24 weeks. We generated 16S rRNA gene (16S) and whole metagenome sequence (WMS) data using stool samples collected from a cohort of 70 women from a randomized, double-blind, placebo-controlled, multicenter clinical trial [18]. Our work provides a novel view of the influence of long-term cranberry consumption on the gut and the possible role of *Flavonifractor* in recurrent UTI disease.

## Results

### Large-scale patterns in bacterial populations unchanged with cranberry consumption

**Fig. 1:**
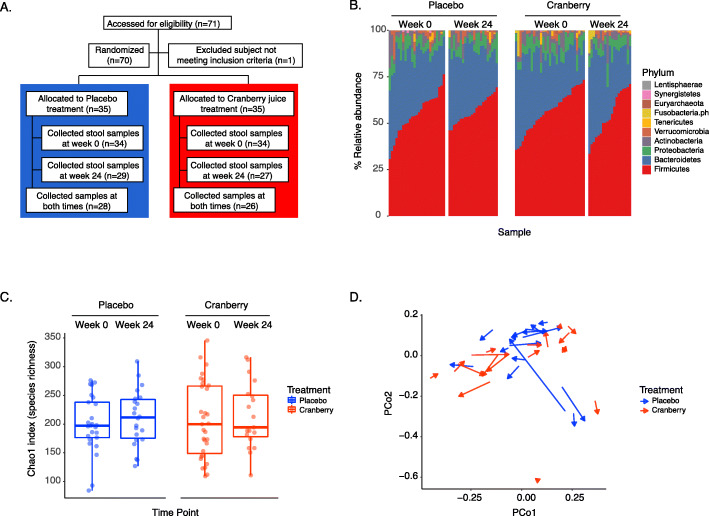
Cranberry beverage consumption does not change the overall composition of the gut microbiome. **a** The cohort consisted of 70 women with a history of recurrent UTIs, who either consumed cranberry beverage or placebo daily for 6 months. The stool samples, collected before and after the 6-month study period, were subjected to 16S rRNA and whole metagenomic shotgun sequencing to infer gut microbial profiles and functions. **b** 16S rRNA-based taxonomic profiles displaying the phylum-level composition of the microbial population indicate that the composition did not change over time or due to cranberry consumption (Wilcoxon rank sum test, *p* > 0.3). The sample order was sorted by the relative abundance of *Firmicutes*. **c** The species richness, based on 16S OTUs, did not change significantly with cranberry beverage consumption (Wilcoxon rank sum test, *p* > 0.6). **d** A comparison of all samples at the 16S OTU level, using principal coordinate analysis (PCoA) based on Bray-Curtis dissimilarities, indicated that the samples from the cranberry cohort did not cluster into a specific group, and the trajectories from week 0 to week 24 (shown by arrows) were scattered, indicating no common shift in microbial composition through time. The magnitude of the change between timepoints, when comparing the two cohorts, was also not significant (*p* = 0.51). The first two principal components (PCo1 and PCo2) accounted for 13.4 and 8.9% of the variability, respectively

As part of a larger clinical study [18], 70 female patients with an average age of 44 (s.d. = 14) years old suffering from frequent rUTI (defined as two or more clinically diagnosed UTIs in the past year, of which at least one UTI had occurred within the past 6 months) were randomly assigned to consume a cranberry beverage (*n* = 35) or placebo (*n =* 35) daily for 24 weeks and provided stool samples at the start (week 0) and end (week 24) of the study window. Fifty-four of these subjects (26 from the cranberry arm and 28 from the placebo arm) provided samples at both time points (Fig. 1a). Total DNA was extracted from stool samples and used to perform 16S rRNA gene (targeting the V4 region) and whole metagenome shotgun (WMS) sequencing (see Materials and Methods).

Using UPARSE to cluster and UTAX [38] to taxonomically classify the 16S data into operational taxonomic units (OTUs), we identified a total of 943 OTUs across all subjects belonging to 172 unique bacterial genera and 10 bacterial phyla. Individual subject samples had a median of 169 OTUs (minimum of 64, maximum of 298). There was no significant difference in the number of OTUs when comparing individuals across the different study arms (Wilcoxon rank sum test, *p* = 0.67) or between their week 0 and week 24 samples (Wilcoxon rank sum test, *p* = 0.51). Similar to other human gut microbiome studies [39], the majority of bacteria across all samples belonged to the phyla *Firmicutes* and *Bacteroidetes*. Neither the phylum-level composition (Fig. 1b) nor the OTU-level composition [40] (*p* > 0.1 for both study arms; see Materials & Methods) of the microbiota changed significantly with time or in response to cranberry or placebo consumption. Diversity metrics were also not significantly different between study arms (Fig. 1c, Fig. S1a) [41, 42].

As seen for other diet-based microbiome studies [43–48], we observed that same-subject samples tended to be more similar to one another than to samples from different subjects (*p* = 1.6 × 10^− 20^), regardless of which beverage they consumed daily (Fig. S1b), with no apparent clustering of communities based on cranberry beverage or placebo consumption (Fig. 1d). Further, the trajectories of community profiles from cranberry beverage or placebo consumers (shown by arrows in Fig. 1d) from week 0 to week 24 were scattered in their directionality, indicating no common shift in microbial composition after 24 weeks due to either beverage. Similarly, there was no significant difference in the magnitude of changes observed after 24 weeks when comparing the placebo arm to the cranberry arm (Fig. 1d; Fig. S1c), indicating that cranberry consumption did not result in a stronger change of the gut microbiota over time compared to placebo.

Using MetaPhlAn2 [49], a tool that estimates bacterial community structure composition from WMS data, we confirmed results from the 16S-based analysis that cranberry consumption does not change the overall gut composition (Additional file 1; Fig. S2). We also analyzed the WMS data using HUMAnN2 [50], a tool that predicts functional pathway and gene family predictions from WMS data. We searched for pathways as well as gene families that differed significantly in abundance in the overall metagenome between study arms following 24 weeks consumption of a cranberry vs. placebo beverage (see Materials and Methods), but found no pathways or gene families that were significantly different between cranberry and placebo after 24 weeks after multiple hypothesis correction (Additional file 2).

### OTU41, a *Flavonifractor* species, decreased in abundance in the cranberry-consuming group, with an opposing increase in the placebo group

**Fig. 2:**
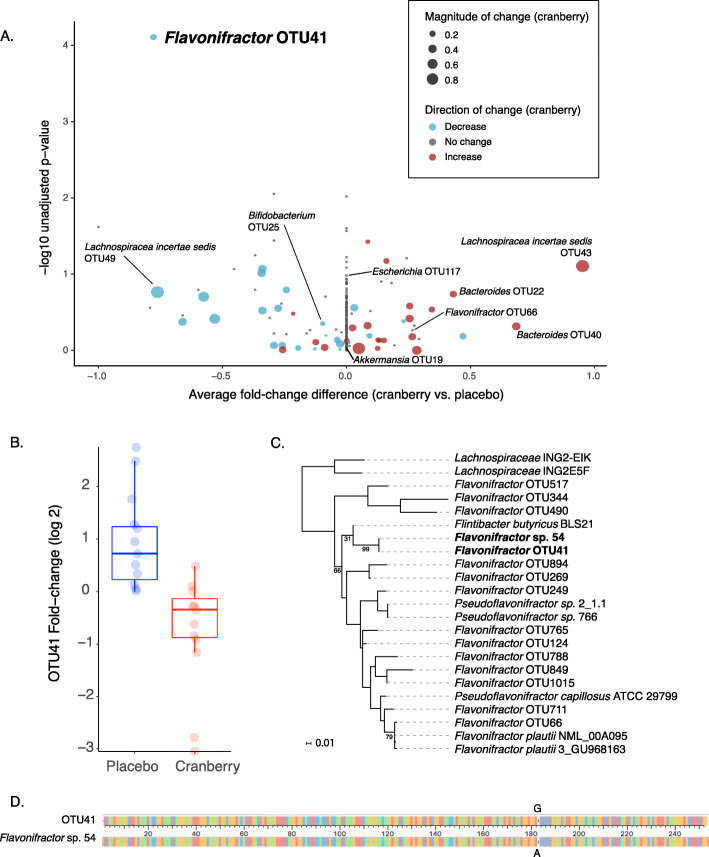
OTU41 is the only significantly different OTU between the cranberry and control cohorts. **a** Distribution of OTU relative abundance differences between cranberry and control cohorts. The X axis represents the difference of the log2 median fold-change of cranberry to placebo cohorts for each OTU. The Y axis is the -log_10_ unadjusted *p*-value of the Wilcoxon rank sum test for each OTU. The size of the point represents the magnitude of the median change in the OTU relative abundance in the cranberry cohort, while the color indicates the direction of said change (blue = decrease, gray = no change, red = increase). Only OTU41 (shown in the top left) was significant after correction (adjusted *p* = 0.02). **b** The relative abundance of OTU41 decreased significantly in the cranberry beverage cohort. **c** A phylogenetic tree, based on 16S V4 regions, of OTU41 together with the other OTUs from this study assigned to the *Flavonifractor* genus, as well as additional sequenced strains closely related to *Flavonifractor*, including *Flavonifractor* sp. 54, *Flavonifractor plautii*, *Lachnospiraceae bacterium*, *Flintibacter butyricus*, *Pseudoflavonifractor* sp., and *Pseudoflavonifractor capillosus* indicated a very close relationship between OTU41 and *Flavonifractor* sp. 54. Relevant bootstrap values are shown. **d** Alignment of the 16S V4 sequence for OTU41 and *Flavonifractor* sp. 54, showing only one base pair difference

Significant changes in individual species have been observed during dietary interventions, even if these changes do not significantly affect the overall composition and/or structure of the gut microbiome communities [44, 46, 51]. To determine whether individual OTUs were affected by cranberry consumption, we examined the change in relative abundance of each of the 219 OTUs present in at least 25% of subject samples in the study (see Materials and Methods), focusing on the more common and consistently present OTUs. Overall, across the vast majority of OTUs tested, we observed no significant difference in the relative changes of OTU abundances across study arms, even before multiple hypothesis testing correction (Fig. 2a). This included OTUs representing bacterial species previously shown to be affected by cranberry in in vitro or mouse models, including *Bifidobacterium longum* [37] and *Akkermansia muciniphila* [33, 34] (Fig. S3). *E. coli* was found at low abundance (mean of 0.05% relative abundance, range 0–0.8%) and its relative abundance was also not significantly affected by cranberry vs. placebo consumption (Fig. S3). WMS data analyzed with MetaPhlAn2 (Additional file 1) and HUMAnN2 (Additional file 2) confirmed that these species, as well as their associated gene functions and pathways, were not significantly different, further supporting the limited effects on both taxonomic and functional composition of the gut microbiome by cranberry consumption.

Only one OTU, identified here as OTU41, showed a statistically significant difference between the cranberry and placebo study arms after multiple hypothesis correction. OTU41, detected in 75% of placebo and 67% of cranberry individuals who supplied both week 0 and week 24 samples, was significantly decreased after 24 weeks of daily cranberry consumption relative to the placebo (p_adj_ = 0.02; Fig. 2b). This difference was driven both by an increase in abundance in the placebo arm (median log_2_ fold change = 0.6) and a decrease in the cranberry arm (median log_2_ fold change = − 0.2) after 24 weeks. We validated this result using an orthogonal statistical approach (p_adj_ = 0.02; see Materials and Methods) [52, 53]. Matching WMS data also showed a similar pattern of abundance of exact sequences matching the OTU41 V4 region of the 16S rRNA gene between the cohorts (*p* = 0.03; Fig. S4; see Materials and Methods). The observed difference in abundance was specific to this OTU, and not a feature of the overall genus; notably, analyses of WMS data using MetaPhlAn2 indicated no significant abundance differences in the overall *Flavonifractor* genus (Additional file 1), and HUMAnN2 results indicated no difference in gene families and pathways (Additional file 2) associated with the overall genus, or any *Flavonifractor* species included in their database. OTU41 represents an unnamed species of *Flavonifractor.*

OTU41 was classified by UTAX [38] as a member of the *Ruminococcaceae* family (93% confidence) and the *Flavonifractor* genus (69% confidence). In order to place OTU41 into evolutionary context with other *Flavonifractor* species, we performed a phylogenetic analysis of the 16S V4 regions of all OTUs from our study assigned to the *Flavonifractor* genus, together with sequences of close relatives determined by searching NCBI’s 16S ribosomal database using the OTU41 sequence as a query (Table S1; see Materials and Methods). The resulting phylogeny revealed that the *Flavonifractor* genus was not monophyletic and contained members from the *Flintibacter* and *Pseudoflavonifractor* genera intercalated between various *Flavonifractor* species (Fig. 2c). Notably, OTU41 was quite distant, differing by 13 SNPs (95% similarity) within the V4 region from the type strain of *Flavonifractor plautii*, the most well-characterized species of the genus. While it appeared that study participants harbored *F. plautii* (OTU66 in this study), its relative abundance did not differ significantly between the study arms (Fig. S5).

OTU41’s closest relative with a whole genome sequence available was a representative strain of an unnamed *Flavonifractor* species, *Flavonifractor* sp. 54, which was isolated from human stool [54] (Fig. 2c). The OTU41 16S V4 region differed from *Flavonifractor* sp. 54 by only one SNP (183G > A; Fig. 2d). Oligotyping of our 16S data (see Materials and Methods) showed that OTU41 contained two major alleles at position 183 (Fig. S6a), one of which was an exact match to *Flavonifractor* sp. 54 and both of which increased in abundance in the placebo arm and decreased in the cranberry arm over the course of the trial (Fig. S6b).

To assess the degree of similarity between the genome sequence of *Flavonifractor* sp. 54 and that of OTU41 members present in our cohort samples, we individually aligned the WMS data from each sample in our study to the reference assembly of *Flavonifractor* sp. 54 and identified individual positions that differed between *Flavonifractor* sp. 54 and similar sequence found within each sample. Between 7 and 85% of the *Flavonifractor* sp. 54 genome was covered by WMS reads, depending on the sequencing depth of each sample and its predicted OTU41 relative abundance. Using samples for which at least 50% of the *Flavonifractor* sp. 54 genomic positions were confidently covered by WMS data, we determined that OTU41 had a median average nucleotide identity (ANI) of 99.3% (range 97.3 to 99.7%) compared to the *Flavonifractor* sp. 54 genome. The current genomic-based definition of a bacterial species places the cutoff for inclusion within a species at 95% ANI [55] (Kim et al., 2014), which suggests that OTU41 representatives from this study and *Flavonifractor* sp. 54 are all members of the same species.

**Fig. 3:**
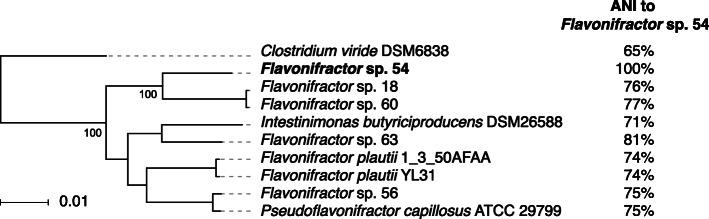
Whole-genome comparative analysis of *Flavonifractor* genomes related to *Flavonifractor* sp*.* 54. Genome sequences were selected based on phylogenetic proximity to *Flavonifractor* sp. 54 and used to construct a whole-genome phylogeny using TBA. All bootstrap support values are 100%. Average Nucleotide Identity calculations indicated that *Flavonifractor* sp*.* 54 is in its own separate species from other references (< 95% ANI is considered a separate species)

Due to OTU41 being only a minor component of the metagenome of these subjects, and our inability to access primary stool specimens for culturing, we could not generate a high-quality assembly of the OTU41genome from any of these subjects. Thus, we used the previously sequenced *Flavonifractor* sp. 54 genome as a proxy for the OTU41 genome. First, to further understand the evolutionary relationship between OTU41 and other whole genome sequenced organisms, we constructed a high-resolution phylogeny based on whole genome alignment of the *Flavonifractor* sp. 54 genome to close relatives that had whole-genome sequences available at NCBI, including (i) four additional *Flavonifractor* isolates [54], (ii) four related species’ type strains, and (iii) *Clostridium viride* as an outgroup (Fig. 3, Fig. S7; Table S2; see Materials and Methods). Consistent with the 16S V4-based phylogeny, the whole genome-based phylogeny showed that *Flavonifractor* sp. 54 was distantly related to *F. plautii* (74% ANI). Although *Flavonifractor* sp. 54 was most closely related in our whole genome phylogeny to other *Flavonifractor*-related organisms previously isolated from human stool [54], ANI calculations indicated they were from different species (81% ANI comparing *Flavonifractor* sp. 54 and *Flavonifractor* sp. 63, the closest isolate genome sequenced).

### Predicting characteristic functional capabilities of OTU41

We next sought to understand why OTU41 responded to daily consumption of the beverages, and to connect changes in abundance of OTU41 with observed differences in clinical presentation of UTI. The tool HUMAnN2 relies on previously generated genomic and gene family databases [49, 50]; given that the closest reference species to OTU41 was *F. plautii*, with only 74% ANI to *Flavonifractor* sp. 54, HUMAnN2 was unable to identify gene families and pathways, including those taxonomically assigned to *Flavonifractor*, that were significantly different between the cranberry and placebo arms after 24 weeks. Thus, we took a more targeted approach, using a combination of comparative genomics and comparative metagenomics, to identify potential special functional capabilities of OTU41. We defined these special functions or “OTU41-characteristic genes” as those found in the *Flavonifractor* sp. 54 genome and in metagenomic samples containing abundant OTU41, but not found in other *Flavonifractor* or related genomes or in metagenomic samples that did not contain detectable OTU41.

To identify “OTU41-characteristic genes”, we first used gene orthology estimates (see Materials and Methods) to compare the gene content of *Flavonifractor* sp. 54 to the whole genome sequences included in our comparative analysis (shown in Fig. 3). Of the 4006 total annotated genes in *Flavonifractor* sp. 54, 1579 (39%) were uniquely found in *Flavonifractor* sp. 54 (i.e., no genes from other genomes were contained within the same ortholog cluster), with 66% of these genes assigned some function (Additional file 3; Material and Methods). Second, we hypothesized that the key functions underlying OTU41’s relationship with cranberry/placebo beverage consumption and UTI symptoms would be conserved across all OTU41 organisms across all subjects. Thus, we sought to define the “core” gene set for OTU41 (i.e., genes shared across all members of the species) by aligning our WMS sequencing data to the panel of reference genomes used in the comparative analysis (see Materials and Methods). In cross-referencing these results to the set of unique genes from *Flavonifractor* sp. 54 based on our comparative genomic analysis, we identified 117 “OTU41-characteristic genes” or genes universally and exclusively found in OTU41-abundant samples (i.e., present by WMS alignments in 100% of samples with ≥0.9% relative abundance of OTU41 based on 16S data and absent in all OTU41-negative samples; Additional file 3).

Of these 117 OTU41-characteristic genes, 30 (25%) were annotated as transporters, which was a significant enrichment compared to the prevalence of transporters in the genome as a whole (9.8%; Fisher’s Exact Test, *p* = 3 × 10^− 5^). These OTU41-characteristic transporter genes included: (i) six members of the Tripartite ATP-independent periplasmic (TRAP) transporter family, which are involved in the uptake of organic acids [56], including dehydroascorbate (an oxidized form of ascorbic acid, i.e., vitamin C), (ii) three ABC transporters, including one that likely acts on cobalamin (i.e., vitamin B_12_) (FS27_003216), (iii) two multi antimicrobial extrusion protein (MATE) family efflux transporters and two resistance-nodulation-division (RND) transporters, both of which can mediate resistance to multiple antimicrobials [57, 58], and (iv) two members of the SNF sodium-neurotransmitter symporter family, which are involved in importing various neurotransmitters (e.g., GABA, choline, and monoamine neurotransmitters, such as epinephrine and serotonin) into the cell [59], including one that putatively acts on tryptophan.

Another 23 OTU41-characteristic genes were associated with metabolism, also representing a significant enrichment compared to the number of genes involved in metabolism across the genome as a whole (35% of OTU41-characteristic genes vs.17% of the entire genome, Fisher’s Exact test *p* < 10^− 5^), in line with other bacterial comparative genomics studies showing metabolism as a driver of species diversification [60–62]. OTU41-characteristic metabolic genes included those affecting amino acids (*n* = 8), nucleotides (*n* = 3), carbohydrates (*n* = 6), coenzymes (*n* = 4), and lipids (*n* = 1) (Additional file 3). Among these genes, several were predicted to function in the biosynthesis or transport of cobalamin, a vitamin that, among other effects on microbes, has been shown to increase growth and virulence in *E. coli* [63–66]. These included *cbiK* (sirohydrochlorin cobaltochelatase, FS27_003212), *cbiE* (precorrin-6y C5,15-methyltransferase, FS27_003540) and *btuC* (permease, FS27_003216), within the BtuCDF Vitamin B_12_ ABC transporter complex [67]. Providing further evidence that OTU41 can synthesize cobalamin, the *Flavonifractor* sp. 54 proxy genome contained two operons for cobalamin synthesis from the de novo anaerobic and salvage pathways [68] (Additional file 3, operons 659 and 1521). Although these complete operons did not meet our strict criteria for OTU41-characteristic genes, further inspection of WMS data by alignment suggested these genes were generally present in OTU41-abundant samples (Fig. S8).

## Discussion

We investigated the effects of cranberry beverage consumption on the human gut microbiome in a 24-week long clinical trial involving women with a history of recurrent UTI. In this trial, women who drank a cranberry beverage daily had a 41% reduction in UTI symptoms as compared to women drinking a placebo beverage [18]. Previous works have proposed that cranberry: i) has an inhibitory effect on *E. coli* [25–30, 69], the leading cause of UTI; ii) can potentially reduce inflammation [35, 36]; and iii) may have anti-*E. coli* or anti-inflammatory/antioxidant effects in the bladder [23, 24, 31, 32]. Other studies have reported that cranberries boost “beneficial” gut bacteria such as *Bifidobacterium longum*, believed to inhibit the growth of pathogenic organisms [37], and *Akkermansia muciniphila*, which have been associated with reduced intestinal inflammation [33, 34].

Our analysis of 16S and WMS profiles generated from the stool microbiota of 70 women who participated in this trial showed no significant differences in the overall diversity or composition (taxonomic or gene-based) of the gut microbiome of women who drank cranberry versus a placebo beverage daily for 24 weeks. Similarly, there was no significant difference in the relative abundances of gut *Bifidobacterium longum*, *Akkermansia muciniphila*, or *E. coli*. The latter result was consistent with results from the larger trial which found that, although symptoms of UTI decreased significantly in cranberry consumers, there was no significant difference in the number of microbiologically positive UTIs or the presence of *E. coli* in the urine [18]. It is also possible that our study did not have the power to detect subtle differences in microbiome composition due to its relatively small sample size. We confirmed this with a post-hoc power analysis [70], which estimated we only had between 5 and 12% power to detect changes in various OTUs. Further, the effects of multiple hypothesis correction used in analysis of the number of OTUs detected also contributed to our limited statistical power.

The only significant difference that we could detect between study arms was in the relative abundance of an OTU assigned to the *Flavonifractor* genus, OTU41. OTU41 was significantly decreased in relative abundance in cranberry vs. placebo consumers. This difference was driven both by an increase in abundance in the placebo arm and a decrease in the cranberry arm. Intriguingly, the *Flavonifractor* genus got its name from its ability to degrade flavonoids [71, 72], including the proanthocyanidins (PACs) contained in cranberry, which are thought to have an inhibitory effect on uropathogenic *Escherichia coli* (UPEC) [8]. If this *Flavonifractor* species were able to thrive by degrading and/or utilizing PACs present in the cranberry, we would expect it to increase in abundance in the cranberry cohort; however, this was not consistent with our observations, as the relative abundance of OTU41 increased in the placebo over the course of the study. It is not clear why OTU41 levels differed in the cranberry versus placebo arms of the study. However, OTU41 was quite different from the well-characterized *Flavonifractor*, with 74% ANI relative to *Flavonifractor plautii*, indicating that it is a separate species, and undoubtedly encodes distinct functions. While it appeared that study participants harbored *F. plautii* (OTU66 in this study), its relative abundance did not differ significantly between the study arms (Fig. S5).

Recent research exploring the interactions between the gut microbiota and the host nervous system, called the “gut-brain axis”, suggests that the microbial inhabitants of the gut and their metabolites can shape and profoundly alter these critical host systems [73–76]. Members of the *Flavonifractor* genus have been repeatedly reported as associated with a range of human health disorders, including various mental health disorders [77–79], autoimmune disease [80], and poor diet and obesity [81–84]. Collectively, these reports suggest that members of the *Flavonifractor* genus may have a role in modulating the gut-brain axis and may be significantly affected by dietary interventions. Though we observed a broad range of OTUs belonging to the *Flavonifractor* genus in our study, none shared the same patterns of change across placebo and cranberry consumers observed for OTU41. When we examined the phylogeny of these OTUs, together with sequences of close relatives from databases, we observed that *Flavonifractor*, *Flintibacter*, and *Pseudoflavonifractor* strains were often mixed within the same phylogenetic clades, indicating that the taxonomy within this portion of the bacterial tree needs attention and that corresponding species names in existing databases may be unreliable. We were unable to ascertain whether the specific *Flavonifractor* species with recently reported health associations corresponded to OTU41.

Though we present no direct evidence that changes in the relative abundance of OTU41 are linked to rUTI symptomatology, we did find that some OTU41-characteristic functions could suggest a relationship between OTU41 abundances and the regulation of a variety of key host functions, including the transport of tryptophan and cobalamin metabolism. Tryptophan, as well as products of the tryptophan catabolic pathway, are involved in modulating a variety of host functions, including the nervous and immune systems [85–94], and have been implicated in the gut-brain axis [71, 95]. Tryptophan metabolites are also involved in UPEC pathogenesis [96–99] and have also been previously shown to be elevated in the urine of a pediatric cohort of symptomatic UTI patients [100], suggesting tryptophan metabolism may be important in the context of recurrent UTI. The OTU41-characteristic genes also included several genes predicted to function in the biosynthesis or transport of cobalamin, which has been suggested to be a key regulator of the gut microbiome [101] and critical in regulating host-microbial interactions [102]. Importantly, UPEC is only able to use the ubiquitous, host-derived metabolite ethanolamine (EA) in the presence of cobalamin. EA consumption is an important pathway for *E. coli* virulence and pathogenesis through both acting as a nitrogen source to outcompete other microbes and by increasing expression of virulence genes [63–66]. Further characterization of OTU41 would provide insight into its potential modulation of these molecules and their associated pathways.

One noteworthy limitation of our study was that we could not determine gene expression levels for OTU41, or other taxa, across individuals or study arms. Gut microbial gene expression patterns have uncovered important roles of the microbiota in the context of diseases, like IBD [103]. Also, transcriptional studies of *E. coli* in mouse models of UTI have shown that *E. coli* expression profiles, prior to entering the urinary tract, are key to colonization and infection [104]. Thus, it is possible that cranberry-derived compounds present in the gut, and possibly the bladder, could disrupt critical steps in UPEC pathogenesis by altering gene expression and/or other mechanisms [1, 105, 106], but these alterations would not be detected in our analysis focused solely on metagenomic, and not metatranscriptomic data.

Other limitations included that our OTU-level analysis would miss more complex and collective interactions of the gut microbial community that have been recently described [107]. These microbe-microbe interactions may illuminate important processes occurring in the gut community that individual changes in OTUs or bacterial species would not. Also, as we did not have an assembled genome of OTU41, we were limited in our reliance upon the *Flavonifractor* sp. 54 genome to provide us with a proxy for comparison to OTU41 in subject samples. Our metagenomic alignments to this reference revealed that OTU41 shared, at most, 85% of genes in the *Flavonifractor* sp. 54 assembly, indicating that, like most bacterial species, this unnamed *Flavonifractor* species is genomically variable, which makes reference-based inferences of gene content across members suboptimal. Though reconstructing OTU41 genomes from metagenomes was an option, the low (< 1%, on average) relative abundances of OTU41 in these samples made this impractical. Finally, by using stringent thresholds to define OTU41-characteristic genes, including requiring them to be core to OTU41, we may have ignored important genetic factors in OTU41 that could be critical for understanding how this species interacts with its host and the observations in the larger study.

## Conclusion

Our work suggests that long-term daily cranberry consumption elicits no large-scale taxonomic or functional changes to the gut microbiome, yet was associated with a decreased level of *Flavonifractor* OTU41 in the gut compared to long-term consumption of a placebo. We observed that OTU41 harbored various characteristic functions involved in transport and metabolism of various compounds, including tryptophan and cobalamin, important molecules in biochemical pathways that may play a role in UPEC pathogenesis and/or rUTI disease. In the larger study from which these samples were derived, cranberry consumption was associated with a decreased incidence of UTI symptoms, but whether OTU41 played a role in this is unclear. This work adds to a growing body of data associating negative human health effects with increased abundance of *Flavonifractor* in the gut [73–75, 80–84]. Particularly, as rUTIs are increasingly difficult to treat due to rising rates of antimicrobial drug resistance [10, 11, 108], further characterization of cranberry products and their role in modulating the gut microbiome, including their effects on OTU41 and possible resident *E. coli*, will help to reveal their effects on rUTI presentation and outcome, and may also provide insight into desperately-needed alternative, non-antibiotic treatments for rUTI.

## Materials and methods

### Cohort study

We used results from a previously published cohort study [18]. This study was registered at clinicaltrials.gov as NCT01776021. This 24-week, double-blind, randomized, placebo-controlled trial on (otherwise) healthy women with a history of rUTI was designed to assess the effect of cranberry beverage consumption on the human gut microbiome. The study was conducted at seven clinical research sites between February 2013 and March 2015. The protocol was approved by an institutional review board in the United States (Quorum Review IRB, Seattle, Washington) and by the National Security Agency for Medicines and Health Products and an Ethical Research Committee (Committee for Personal Protection) in France. Procedures were followed in accordance with the Declaration of Helsinki of 1975 as revised in 1983. Written informed consent was obtained from all subjects. The eligible subjects were 20–70 year-old women with a recent history of UTI and BMI < 40 kg/m^2^. Women were not enrolled if they were using prophylactic antibiotics for a UTI. Detailed subject inclusion criteria have been previously published [18]. Fecal samples were collected at week 0 and week 24, as well as at the time of any additional UTIs during the study. Metadata for each provided stool sample can be found in Additional file 4.

### 16S rRNA sequencing and analysis

In order to infer the microbial composition at the species level, we sequenced the 16S rRNA V4 region for all 131 samples using the Illumina MiSeq platform. Our DNA extraction protocol included steps involving bead beating and heating in order to expand the range of microbial species which could be observed [109]. Our 16S rRNA sequencing targeted amplification of a ~ 250 bp region of the microbial 16S rRNA gene with tailed primers (515F & 806R) to generate 175 bp paired-end reads [110]. The mean read depth per sample was 152,300 paired reads. Reads are publicly available on NCBI BioProject PRJNA528960.

16S rRNA sequencing reads from 131 samples were analyzed using UPARSE [38] with version 14 of the RDP database [111], using a 97% identity threshold. We used UPARSE to perform quality filtering of reads, discarding singleton reads and then clustering the remaining reads into operational taxonomic units (OTUs). Representative sequences for OTUs in FASTA format can be found in Additional file 5. UPARSE generated an OTU matrix containing 1073 OTUs from 131 samples (Additional file 6). The 7 samples that were collected at time of UTI were excluded from downstream analysis, leaving 1070 OTUs from 124 samples. Rarefaction analysis was performed with the q2-diversity plugin in QIIME2 [52]. Read depths from 1000 to 20,000 reads per sample were tested, and average Chao1 vs. the number of samples retained was plotted. A value of 5000 reads per sample was chosen for downstream analysis, as it resulted in high Chao1 values (~ 75% of highest depth tested) while retaining most samples (~ 80% of samples). Then, to normalize for uneven sequencing depth across samples, we rarefied the OTU read counts of each sample to 5000 reads, resulting in an OTU matrix with 1023 OTUs from 97 samples. We additionally filtered out OTUs with fewer than 10 reads total in order to remove rare and ultra-low abundant OTUs, which we would not have enough power to detect differences in [112], resulting in 943 final OTUs across 97 samples for analysis (Additional file 7). Taxonomic assignment of each OTU was performed using the UTAX algorithm, a k-mer based method that looks for common k-mers between query and reference sequences with known taxonomy and assigns confidence estimates based on data training, using the RDP v16 database training set provided by the UPARSE author. To assign species level to OTUs of interest (Fig. S3), representative sequences were queried against the NCBI 16S ribosomal RNA database using blastn (megablast, default parameters).

In order to test for OTU-level compositional differences before vs. after treatment across both study arms, we used a generalized Hotelling’s test (GHT) implemented in R [40]. In brief, the GHT tests for whether the average microbiome compositions of paired samples (i.e., before and after the study) are the same or different. We ran the GHT for each study arm independently.

The OTU matrix was used to calculate the α-diversity (species richness) using the Chao1 index [41] and the Shannon diversity index [42], as well as the β-diversity (species dynamics) using the Bray-Curtis dissimilarity [113]. All of these metrics were implemented in v1.8 of QIIME [114].

### Statistical analysis to identify significantly different OTUs

In order to determine if OTUs were significantly different between cranberry and placebo, we performed Wilcoxon rank sum tests on the log_2_ fold-changes of each subject, comparing week 24 to week 0 read counts, calculating whether the cranberry group significantly differed from the placebo group on a per-OTU basis. This method accounts for differences between individuals’ microbiomes prior to starting the treatment, calculating independently how each individual changed over time. Prior to calculating fold-change, the rarefied OTU Table (5000 reads per sample) was filtered to exclude subjects that did not have samples from both week 0 and week 24, as well as to exclude OTUs that were present (one or more 16S reads) in fewer than 25% of subject samples (from both arms from both week 0 and 24), which reduced the number of OTUs analyzed from 943 to 219 and the number of subjects considered to 16 from placebo (32 paired samples) and 18 from cranberry (36 paired samples). Fold-change values were calculated as follows: log_2_((reads_wk24_ + 1) / (reads_wk0_ + 1)), accounting for patients that had no evidence of the OTU in either time point such that they had a fold-change of 0. *P*-values from the Wilcoxon rank sum tests were adjusted for multiple hypothesis correction using Benjamini-Hochberg [115]. We also assessed different thresholds for OTU inclusion in the analysis, including being present in 5, 10, and 50% of subject samples, but for all thresholds tested, OTU41 was consistently the only significant result after *p*-value correction (data not shown).

To validate OTU-level differences using an orthogonal approach, we ran the pairwise-differences test from the q2-longitudinal plugin implemented in QIIME2 [52, 53] for each filtered OTU independently. In brief, the rarefied OTU count table was imported into QIIME2 and transformed into relative frequencies. For each OTU, we ran the pairwise-differences script and reported the *p-*value for the pairwise differences test. We corrected reported *p*-values with Benjamini-Hochberg.

### Oligotyping analysis of OTU41 16S rRNA sequence data

Oligotyping was performed on 16S rRNA sequencing data using the Python oligotyping pipeline (http://merenlab.org/software/oligotyping/) [116, 117]. Briefly, we selected the subset of reads assigned to OTU41 by the UPARSE algorithm (i.e., reads within 97% identity OTU41 consensus sequence), aligned them to the OTU41 consensus sequence, and performed oligotyping analysis, which identifies nucleotide positions that represent information-rich variation among these sequences and then assigns oligotypes based on those varying sites. This method distinguishes real biological variation from sequencing errors through calculating entropy of each position in the alignment [117]; sequencing errors should be generally randomly distributed along the length of the alignment, appearing as “white noise” in the entropy profile. The authors of the tool set a recommended entropy cutoff of 0.2. The highest site of variation within our 16S data was at position 183, the same position that differed from the *Flavonifractor* sp. 54 reference sequence, which had an entropy of approximately 1. We opted to include only this site in oligotyping, as other sites had comparatively little variation (entropy between 0 and 0.4; Fig. S9). Oligotyping revealed three alleles at position 183: 183G, 183A, and 183 T, which represented 65, 34 and < 1% of all sequences, respectively.

### 16S rRNA phylogenetic analysis

16S rRNA sequences included in our analysis are listed in Table S1. We included the centroid sequences from the UPARSE pipeline to represent each of the OTUs assigned to the *Flavonifractor* genus using the UTAX algorithm with at least 40% confidence (see above). We also used blastn to search NCBI’s 16S ribosomal RNA database using OTU41 as a query. Sequences from the top hits were included. Sequences of two strains of *Lachnospiraceae* bacterium were included as an outgroup.

The online 16S aligner from SILVA, SINA (v1.2.11) [118], was used to align these 16S rRNA sequences. The alignment was filtered with Mothur (v.1.36.1) [119] to retain only the V4 region. A phylogenetic tree was constructed using RAxML (v7.7.8) [120] using the GTRCAT model with 100 rapid bootstraps (9 out of 39 branches had ≥50% bootstrap support values). The resulting tree was rooted with the *Lachnospiraceae* outgroup strains.

To generate the 16S phylogeny containing both OTUs from this study and the reference genomes, the annotated 16S rRNA genes were extracted and aligned as above, and the V4 region was retained. These alignments were used to generate a phylogenetic tree, as above, rooting the tree using *Clostridium viride* as an outgroup.

### Whole-metagenomic shotgun sequencing and analysis

Using the same 131 extracted DNA samples that we used for 16S rRNA sequencing, we constructed metagenomic libraries using the Nextera XT library prep kits (Illumina, San Diego, CA) and sequenced the total genomic DNA content of each sample on the Illumina HiSeq 2500 platform with paired-end 100 bp reads. We obtained a median read depth of 870,144 and a mean of 909,759 paired reads (range 46,508-1,696,354).

Taxonomic profiling was performed using MetaPhlAn2 [48], which unambiguously classifies metagenomic reads to taxonomies based on a database of clade-specific marker genes derived from 17,000 microbial genomes (corresponding to > 7500 bacterial, viral, archaeal and eukaryotic species). The resulting taxonomic profiling matrix was used to calculate the α-diversity (species richness) using the Chao1 index [41] and the β-diversity (species dynamics) using Bray-Curtis dissimilarity [113].

Reference genomes from the species identified by MetaPhlAn2 were used to perform functional profiling using HUMAnN2 (version 0.9.6) [49, 50]. Briefly, whole-metagenomic shotgun reads were mapped using Bowtie2 [121] to sample-specific reference genomes, including all gene families in any microorganism present. For the reads that were not mapped by Bowtie2, a further translated search using DIAMOND [122] was then performed against UniRef50 [123]. Hits were counted per gene family and normalized for length and alignment quality. Gene family abundances from both the nucleotide and the translated searches were then combined into structured pathways from MetaCyc [124] with MinPath [125] with the gap filling options to generate a functional profiling matrix, which was normalized per sample to relative abundance values

Analysis was performed on HUMAnN2 data for each pathway and each gene family, similar to the 16S methods. Data were filtered to remove less abundant pathways and genes, retaining pathways and gene families that were represented in at least 50% of patients from each study arm, retaining 1651 (out of 9290) pathways and 415,257 (out of 2,392,193) gene families. No read count or relative abundance filtering was performed on pathways or gene families. Wilcoxon rank sum tests were performed per pathway/gene family on the fold-change values from each subject (i.e.*,* log2(week 24 / week 0)), adding a small number to both numerator and denominator to prevent log of zero and divide by zero errors (0.001 for pathways and 0.0001 for gene families), comparing cranberry subjects to placebo subjects. *P*-values were adjusted using Benjamini-Hochberg [115].

To obtain relative abundance of the OTU41 V4 region sequence in WMS data, paired reads were aligned with bwa mem (v0.7.12-r1039; https://github.com/lh3/bwa) [126] and reads with identical sequence to the OTU41 sequence were counted per sample. Read counts were normalized based on sequencing depth per sample to relative abundance. A Wilcoxon rank sum test was performed on the fold-changes of relative abundance, comparing week 24 to baseline, to determine whether changes in OTU41 in this datatype were significantly different between cranberry and placebo (*p* = 0.03).

### Alignments of metagenomic samples to *Flavonifractor* sp. *54* assembly to estimate whole-genome ANI between *Flavonifractor* sp. 54 and OTU41

In order to determine the Average Nucleotide Identity (ANI) between sp. 54 and OTU41-like sequence present within each sample, WMS reads were aligned to the *Flavonifractor* sp. *54* genome using bwa mem (v0.7.12-r1039; https://github.com/lh3/bwa) [126], retained only paired reads aligned as proper pairs, with minimum mapping quality of 5, and no more than five nucleotides soft-clipped at the end of the read. We then used the Bcftools (version 1.6; http://samtools.github.io/bcftools/) [127] commands “mpileup”, “call”, and “filter” to determine the number of high quality reference and SNP calls across all 131 WMS samples. For each sample, Bcftools “mpileup” was first used to generate allele information for each position in the *Flavonifractor* sp. 54 reference genome; Bcftools “call” (with ploidy set to 1) was then used to call variants, which were further filtered with Bcftools” filter” to filter out calls with a sum base quality of less than 50. Further, positions were excluded if the depth of high quality calls (i.e., sum of the DP4 field) was 3 standard deviations above the mean depth of passing calls for the sample. This filter was performed to reduce erroneous SNP calls (which would decrease the estimated ANI) from homologous sequences contained in other, more abundant organisms found within each sample.

After applying these filters, the remaining high-confidence, passing SNP calls were then tallied per sample. An estimate of average nucleotide identity (ANI) between the *Flavonifractor* sp. 54 reference genome and the OTU41-like sequence within each sample was calculated by examining the fraction of passing calls that difference from the reference allele, divided by the total number of passing calls present within that sample.

After applying these filters, the remaining high-confidence, passing SNP calls were then tallied per sample. An estimate of average nucleotide identity (ANI) between the *Flavonifractor* sp. 54 reference genome and the OTU41-like sequence within each sample was calculated by examining the fraction of passing calls that difference from the reference allele, divided by the total number of passing calls present within that sample.

### Alignments of metagenomic samples to *Flavonifractor* sp. *54* assembly to compare gene content

In order to compare gene content across the reference genomes in our data set and identify “OTU41-characteristic genes”, reads from each sample were aligned using bwa mem (v0.7.12-r1039; https://github.com/lh3/bwa) [126] to the set of reference genomes used for comparative genomics (Table S2), including *Flavonifractor* sp. 54. Alignments were then filtered for good mapping quality (MQ ≥ 5), properly paired reads, and soft clipping of fewer than 5 bases. To assess gene coverage, the resulting alignments were analyzed using the BEDTools (v2.26.0; https://github.com/arq5x/bedtools2/releases) [128] “coverage” command with our in-house annotation of the *Flavonifractor* sp. 54 reference genome (see below). We considered a gene to be present in a given sample if at least 75% of the length of the gene sequence had at least one read aligned to it. We chose this threshold as it struck a balance by being both fairly sensitive (minimum of one read aligned) and specific (75% of gene covered).

To determine which genes and regions of the *Flavonifractor* sp. *54* reference genome were present in OTU41 (and not likely to be found in related organisms), we used 16S sequencing results to select 19 samples that had ≥0.9% OTU41 relative abundance (i.e. OTU41+) and 44 samples that had < 0.01% OTU41 relative abundance (i.e. OTU41–). We then delineated genes into several categories of increasing specificity/confidence: 1) enriched in OTU41+ samples (Fisher’s exact test, adjusted *p* < 0.05); 2) Highly enriched in OTU41+ samples (covered in ≥18 OTU41+ and ≤ 1 OTU41– samples); 3) Highly enriched genes found in orthogroups exclusive to *Flavonifractor* sp. 54 genome; 4) OTU41 + -specific (covered in all 19 OTU41+ and 0 OTU41– samples); 5) OTU41 + -specific plus only found in orthogroups within *Flavonifractor* sp. 54 genome. Additional file 3 contains results from this analysis.

### Whole-genome comparative analysis

Browne et al. contained multiple sequenced genomes from the *Flavonifractor* genus, including *Flavonifractor* sp. 54 [54]. We included all genomes from this study in the analysis. Then, by using the OTU41 sequence as a query to search against the RDP database, we collected additional related type strain references: two *F. plautii* genomes; *Pseudoflavonifractor capillosus*; and *Intestinimonas butyriciproducens*. We also included *Clostridium viride* as an outgroup for phylogenetic analysis (See Table S2 for a list of genomes included in our whole-genome comparative analysis). Genomes were re-annotated using the Broad Institute’s Prokaryotic Annotation Pipeline [129].

To obtain phylogenies, whole genome alignments were generated using the Threaded Blockset Aligner (TBA v12) [130]. TBA requires an input tree, which was generated using a reconstructed phylogeny generated by FastTree (version 2.1.7) [131] on an alignment of concatenated AMPHORA2 (version 2.0) markers genes [132]. Then, pairwise alignments of all genomes were generated and projected onto the *Flavonifractor* sp. 54 genome, concatenated, and used as input to reconstruct a whole-genome phylogeny using FastTree.

To calculate Average Nucleotide Identities (ANI) between reference genomes, orthogroup clustering was performed using SynerClust (version Nov 13, 2017, https://github.com/SynerClust) [133]. Then, each orthogroup was aligned using MUSCLE (v3.8.31) [134]. Pairwise ANI values were then calculated as the mean of the identical nucleotide positions in the alignment of each common orthogroup between two genomes [135].

### Analysis of 16S rRNA sequence in *Flavonifractor sp. 54* assembly

The raw sequencing reads used to generate the *Flavonifractor* sp. 54 genome assembly were downloaded from the NCBI SRA database (accession number ERR1022445). These reads were aligned back to the reference genome using bwa mem. The coverage of the annotated 16S rRNA gene relative to median coverage of the reference genome was determined and used to estimate the number of copies of this locus in the strain, approximately four copies. Further, Pilon (version 1.22) [136] was used to confirm there is no variation among the four copies of the gene.

### Re-annotation of *Flavonifractor* sp. 54 assembly

To obtain as much functional information related to the gene content of the *Flavonifractor* sp. 54 reference assembly, we re-annotated the genome sequence using the Broad Institute’s Prokaryotic Genome Annotation Pipeline [129], as well as RASTtk (http://rast.nmpdr.org/) [137–139] and DFAST (https://dfast.nig.ac.jp/) [140]. The Broad pipeline also provides KEGG [141], GO [142, 143], TIGRfam [144], and Pfam domain [145, 146] annotations per gene. We additionally ran Operon Mapper to predict genes found within operons (http://biocomputo.ibt.unam.mx/operon_mapper/) [147], which also provided COG [148, 149] and Uniprot [150] information for each gene. Finally, we used BLAST+ blastp [151] with evalue ≤1e-4 to assign eggNOG [152] families to each gene, filtering to retain at least 70% overlap between NOG and alignment length. Gene names were determined using the following priorities: 1) Broad annotation; 2) RAST; 3) DFAST; 4) Uniprot; 5) COG; where the highest priority, non-hypothetical protein annotation was used.

To determine if a gene was a transporter, we searched the gene annotation, COG, GO, and Pfam domain information for the following terms: transport, symport, permease, efflux, pump, and antiport. If a gene and any of its annotations contained at least one of these keywords, it was considered to be a transporter of some kind. We then used Fisher’s Exact test to determine if the 30 putative transporters of the 117 of the OTU41-specific, core genes were significantly enriched compared to the 392 putative transporters of 4006 total genes.

## Supplementary Information

**Additional file 1.** Merged MetaPhlAn2 table from all 131 WMS samples, where each row is a separate taxonomic level. These data have not been normalized.

**Additional file 2. **HUMAnN2 pathway and gene family results. Pathway results are in the first tab, while gene family results are in the second tab. Only gene families with unadjusted *p* < 0.05 are included for brevity. Column B: the average abundance (in counts per million [cpm]) for the cranberry group at week 0; Column C: the average abundance (in cpm) for the cranberry group at week 24; Column D: the average abundance for the placebo group at week 0; Column E: the average abundance for the placebo group at week 24; Column F: the log_2_ ratio of the fold-change over 24 weeks in the cranberry group to the fold-change over 24 weeks in the placebo group, where a positive value indicates a pathway/gene family increased after 24 weeks in the cranberry group relative to the placebo group; Column G: the *p*-value of the Wilcoxon rank sum test comparing the fold-changes after 24 weeks between cranberry and placebo group individuals; Column H: the Benjamini-Hochberg adjusted *p-*value (see Materials and Methods).

**Additional file 3. ***Flavonifractor* sp. 54 gene annotations, including those that were defined as OTU41-characteristic genes. Definitions for columns are found in the first tab.

**Additional file 4.** De-identified sample metadata, which includes sample ID, study arm, anonymous patient ID, and visit information.

**Additional file 5.** FASTA format file containing representative sequences for OTUs in this study, including OTU41.

**Additional file 6.** The unfiltered raw count OTU table generated by UPARSE for all sequenced samples.

**Additional file 7.** The OTU table with sample and OTU filtering, rarefied to 5000 reads/sample, which was used in downstream analysis.

**Additional file 8: Figure S1.** Additional analysis of diversity using 16S data. a) Shannon diversity index, a measure of **ɑ**-diversity, is not significantly different between cranberry and placebo cohort (*p* > 0.5) b) β-diversity of longitudinal samples from the same subject is significantly lower than that between samples from different subjects (*p* = 1.6 × 10^− 20^); c) β-diversity of samples from the placebo cohort versus those from the cranberry cohort do not differ significantly; *p* = 0.51).

**Additional file 9: Figure S2.** Analysis of WMS data confirms that cranberry beverage consumption does not change overall gut microbiome composition. a) WMS-based taxonomic profiles displaying the phylum-level composition of the microbial population indicate that the composition did not change over time or due to cranberry consumption. The sample order was sorted by the relative abundance of *Firmicutes*. b) The species richness, based on WMS data, did not change significantly with cranberry beverage consumption. c) A comparison of all samples at the 16S OTU level, using principal coordinate analysis (PCoA) based on Bray-Curtis dissimilarities, indicated that the samples from the cranberry cohort did not cluster into a specific group, and the trajectories from week 0 to week 24 (shown by arrows) were scattered, indicating no common shift in microbial composition after 24 weeks of cranberry or placebo treatment. The first two principal components (PCo1 and PCo2) accounted for 14.8 and 10.8% of the variability, respectively.

**Additional file 10: Figure S3.** Log_2_ fold changes of OTUs of *Escherichia coli* (OTU117), *Bifidobacterium* sp. (OTU25, likely *B. adolescentis* [OTU consensus sequence is 0 SNPs from *B. adolescentis* reference sequence] or *B. longum* [OTU consensus sequence is 1 SNP from *B. longum* reference sequence]), and *Akkermansia muciniphila* (OTU19). None were significantly different between cranberry and placebo study arms.

**Additional file 11: Figure S4.** Additional analyses further confirm the difference in abundance of the *Flavonifractor* species represented by OTU41 between the cranberry and placebo cohorts, validating our 16S-based OTU-level analysis. WMS read mapping to the OTU41 16S V4 regions confirms that OTU41 differs significantly between the cranberry and placebo cohorts (*p* = 0.03).

**Additional file 12: Figure S5.** The relative abundance of OTU66, the OTU most closely related to *Flavonifractor plautii*, did not change significantly after cranberry beverage consumption (*p* = 0.43).

**Additional file 13: Figure S6.** OTU41 consists of two major oligotypes, 183G and 183A. a) 183G was found in approximately two-thirds of the sequence assigned to OTU41, while 183A was found in approximately one-third, with very minor amounts of 183 T. Both 183G and 183A alleles were represented in women from both study arms at weeks 0 and 24. b) Both major oligotypes of OTU41 behave consistently with the overall behavior of OTU41, trending upwards in placebo and downwards in cranberry consumption.

**Additional file 14: Figure S7.** 16S rRNA V4 region phylogeny containing OTUs from our study, along with sequences extracted from the reference genomes used in the comparative genomics analysis (shown in green). OTU41 and *Flavonifractor* sp. 54 are indicated in bold. Taxonomic assignments for OTUs are shown in brackets, with their confidence value in parentheses. These reference genomes also showed close relationships to OTUs found across our study, indicating the species they represent are also present in study participants, though their relative abundances were unchanged by cranberry or placebo consumption.

**Additional file 15: Figure S8.** WMS alignment results of genes from two cobalamin synthesis operons in the *Flavonifractor* sp. 54 assembly. a) Genomic ANI to *Flavonifractor* sp. 54 vs. gene alignment coverage of each gene. b) Genomic breadth of coverage (in % of genome) vs. gene alignment coverage. c) Average genomic depth of sequencing vs. gene alignment coverage. These results suggest that our choice of a 75% gene coverage threshold for gene presence was conservative as OTU41-containing samples with low OTU41 abundance and/or sequencing depth tended to not meet this threshold despite modest evidence for these genes being present.

**Additional file 16: Figure S9.** Oligotyping entropy profile for OTU41. X axis is the position in the alignment to OTU41 consensus sequence. Y axis is the Shannon Entropy of each position in the sequences that were clustered into OTU41. Position 183 was the only position in the OTU41 alignment that was above an entropy of 0.4. The background highlights the major oligotypes based on the top 6 most entropic positions, which represent the G and A alleles at position 183.

**Additional file 17: Table S1.** 16S rRNA sequences included in phylogenetic analysis. **Table S2.** Whole genomes included in comparative analysis.

## Data Availability

The 16S rRNA and WMS sequencing reads generated and analyzed during the current study are available in the NCBI SRA repository, under the BioProject PRJNA528960 (https://www.ncbi.nlm.nih.gov/bioproject/PRJNA528960). Additional data generated and analyzed during this study are included in this published article and its additional files.
