# Supplementary figures and images for: Limited effects of long-term daily cranberry consumption on the gut microbiome in a placebo-controlled study of women with recurrent urinary tract infections
Source: BMC Microbiol. 2021 Feb 18;21:53. doi: 10.1186/s12866-021-02106-4 (PMC7890861; doi:10.1186/s12866-021-02106-4)

# Figure S1

A.

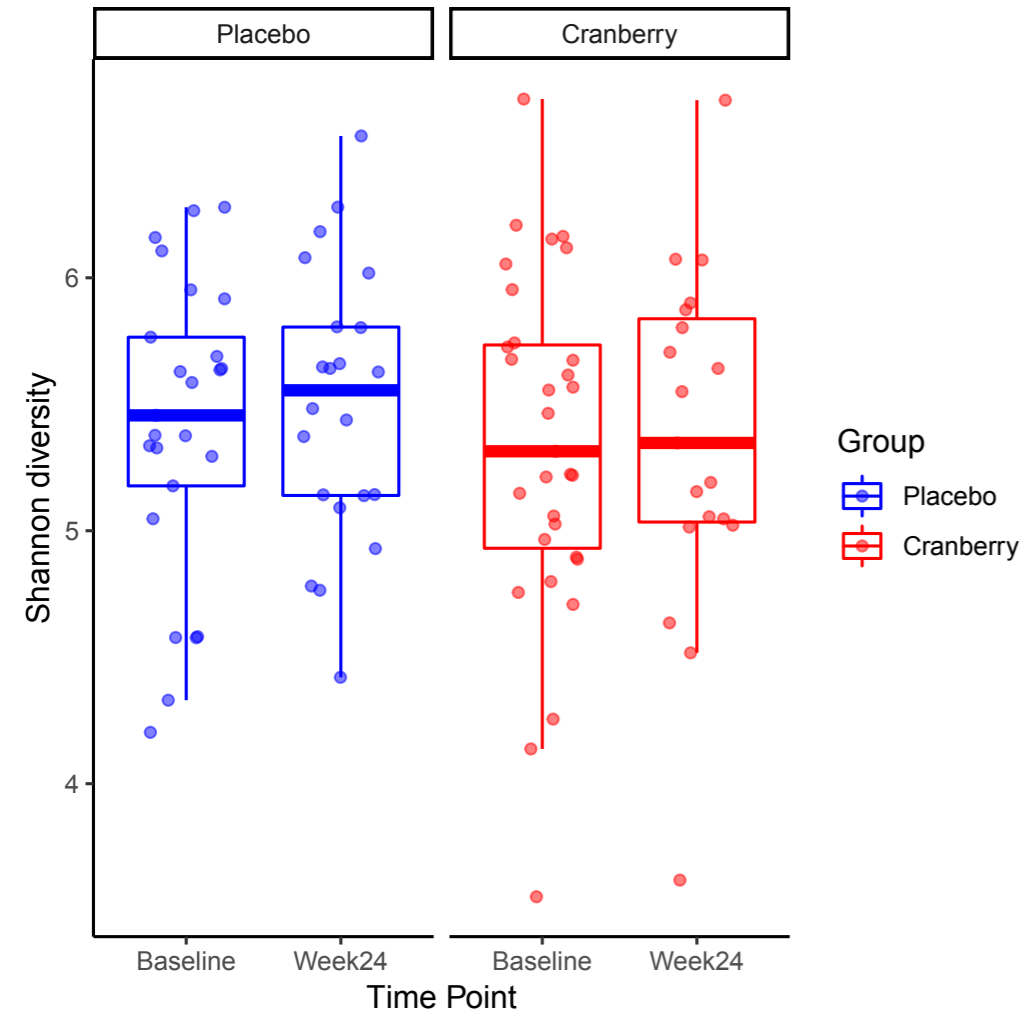

B.

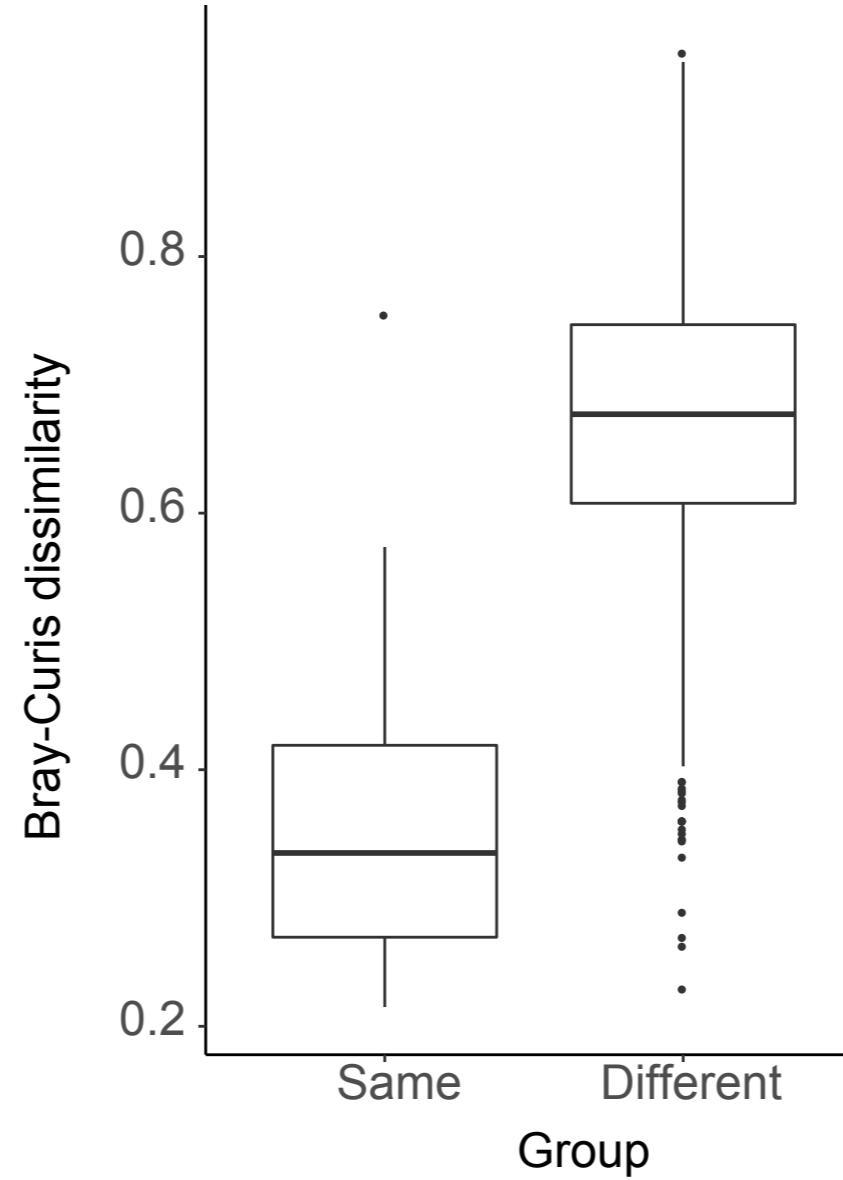

C.

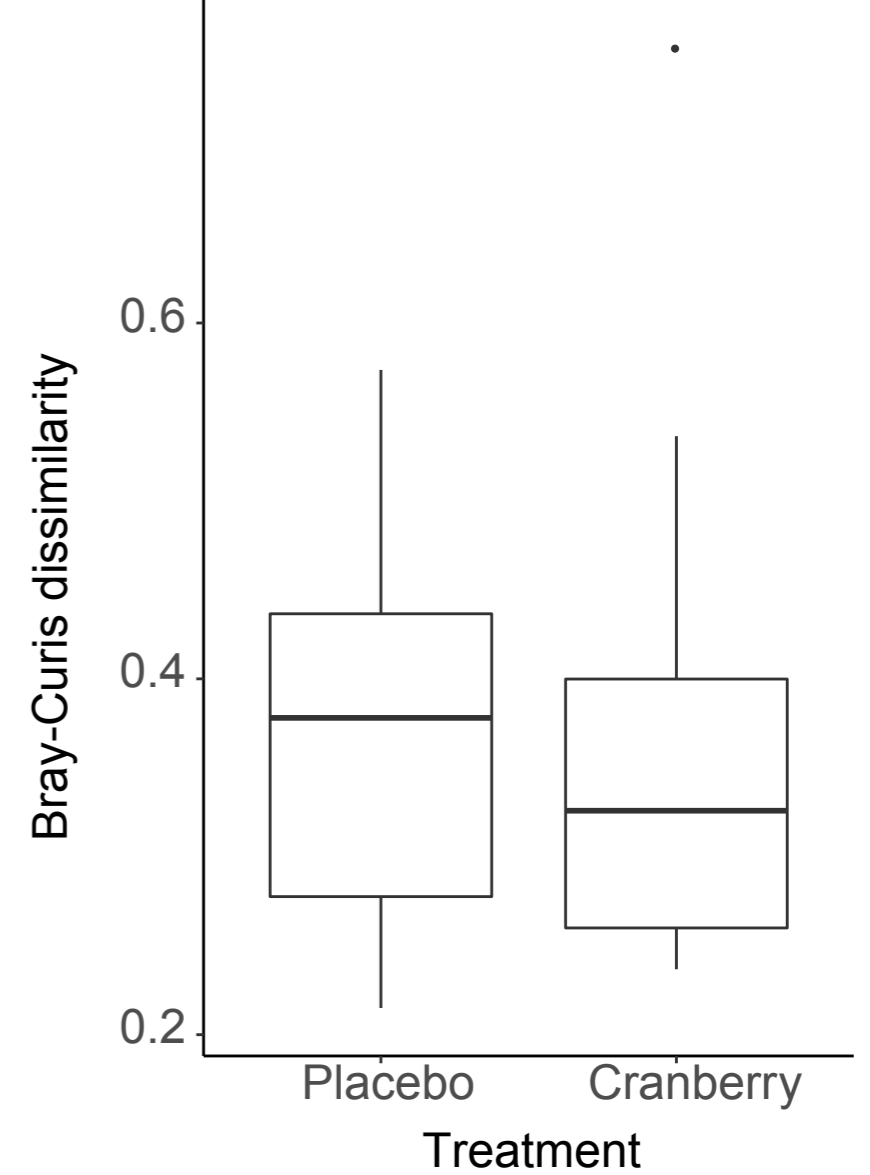

Supplement: Supplementary file 8 — Additional file 8: Figure S1. Additional analysis of diversity using 16S data. a) Shannon diversity index, a measure of ɑ-diversity, is not significantly different between cranberry and placebo cohort (p > 0.5) b) β-diversity of longitudinal samples from the same subject is significantly lower than that between samples from different subjects (p = 1.6 × 10− 20); c) β-diversity of samples from the placebo cohort versus those from the cranberry cohort do not differ significantly; p = 0.51). [file 12866_2021_2106_MOESM8_ESM.pdf]

Figure S2

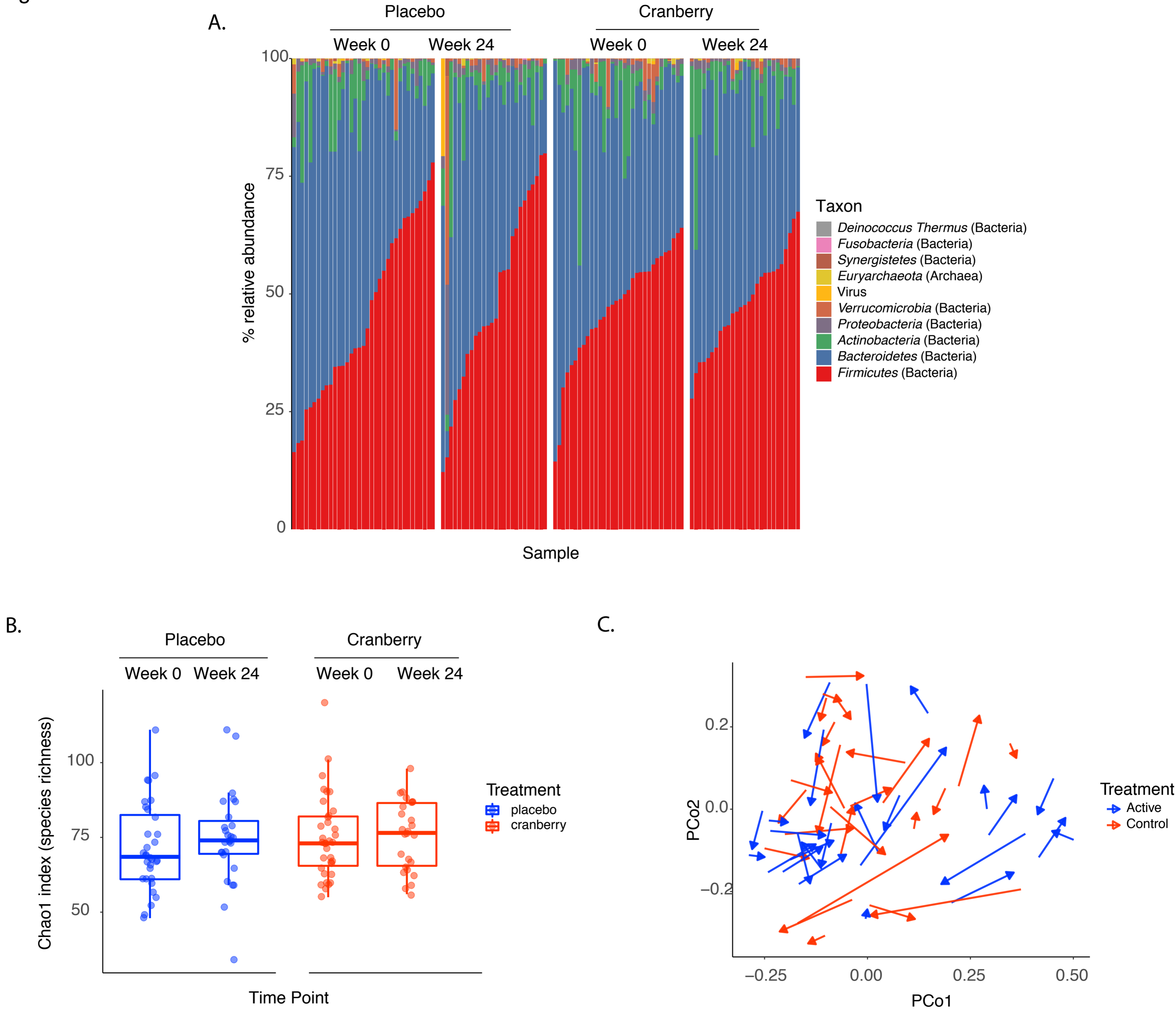

Supplement: Supplementary file 9 — Additional file 9: Figure S2. Analysis of WMS data confirms that cranberry beverage consumption does not change overall gut microbiome composition. a) WMS-based taxonomic profiles displaying the phylum-level composition of the microbial population indicate that the composition did not change over time or due to cranberry consumption. The sample order was sorted by the relative abundance of Firmicutes. b) The species richness, based on WMS data, did not change significantly with cranberry beverage consumption. c) A comparison of all samples at the 16S OTU level, using principal coordinate analysis (PCoA) based on Bray-Curtis dissimilarities, indicated that the samples from the cranberry cohort did not cluster into a specific group, and the trajectories from week 0 to week 24 (shown by arrows) were scattered, indicating no common shift in microbial composition after 24 weeks of cranberry or placebo treatment. The first two principal components (PCo1 and PCo2) accounted for 14.8 and 10.8% of the variability, respectively. [file 12866_2021_2106_MOESM9_ESM.pdf]

# Figure S3

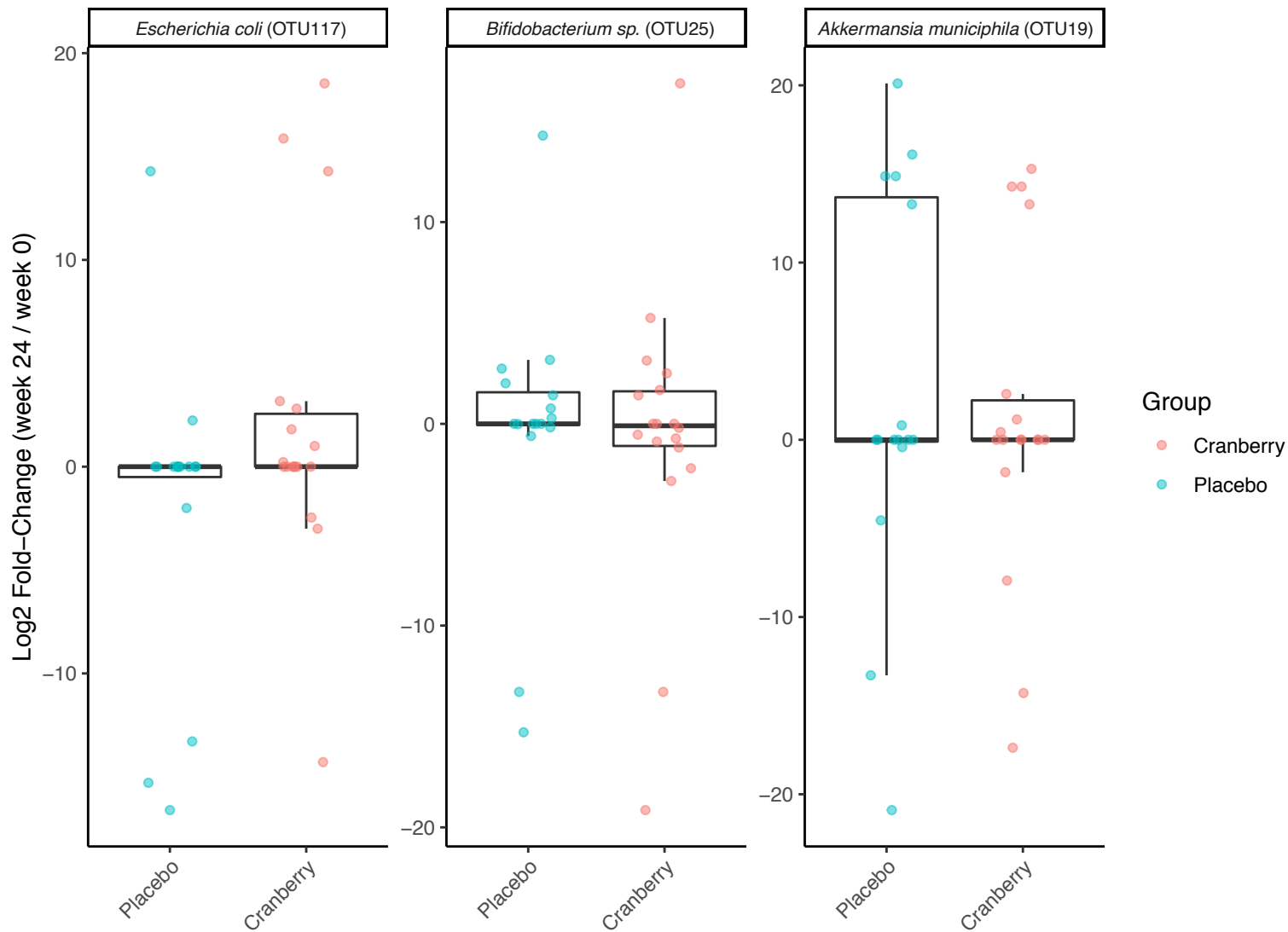

Supplement: Supplementary file 10 — Additional file 10: Figure S3. Log2 fold changes of OTUs of Escherichia coli (OTU117), Bifidobacterium sp. (OTU25, likely B. adolescentis [OTU consensus sequence is 0 SNPs from B. adolescentis reference sequence] or B. longum [OTU consensus sequence is 1 SNP from B. longum reference sequence]), and Akkermansia muciniphila (OTU19). None were significantly different between cranberry and placebo study arms. [file 12866_2021_2106_MOESM10_ESM.pdf]

# Figure S4

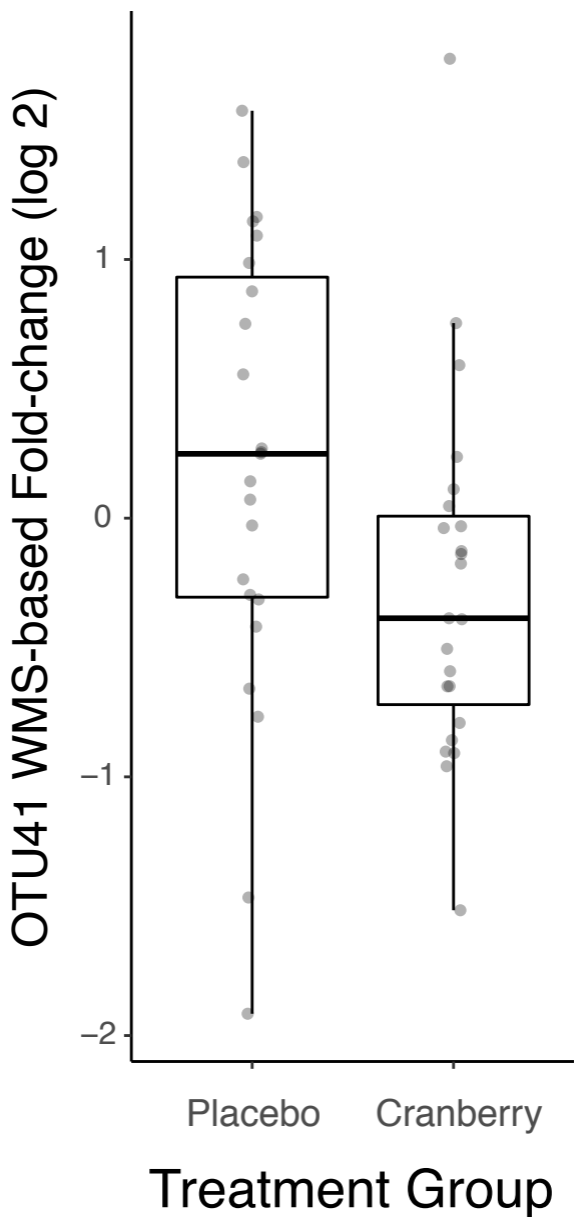

Supplement: Supplementary file 11 — Additional file 11: Figure S4. Additional analyses further confirm the difference in abundance of the Flavonifractor species represented by OTU41 between the cranberry and placebo cohorts, validating our 16S-based OTU-level analysis. WMS read mapping to the OTU41 16S V4 regions confirms that OTU41 differs significantly between the cranberry and placebo cohorts (p = 0.03). [file 12866_2021_2106_MOESM11_ESM.pdf]

# Figure S5

OTU\_66; index: 25

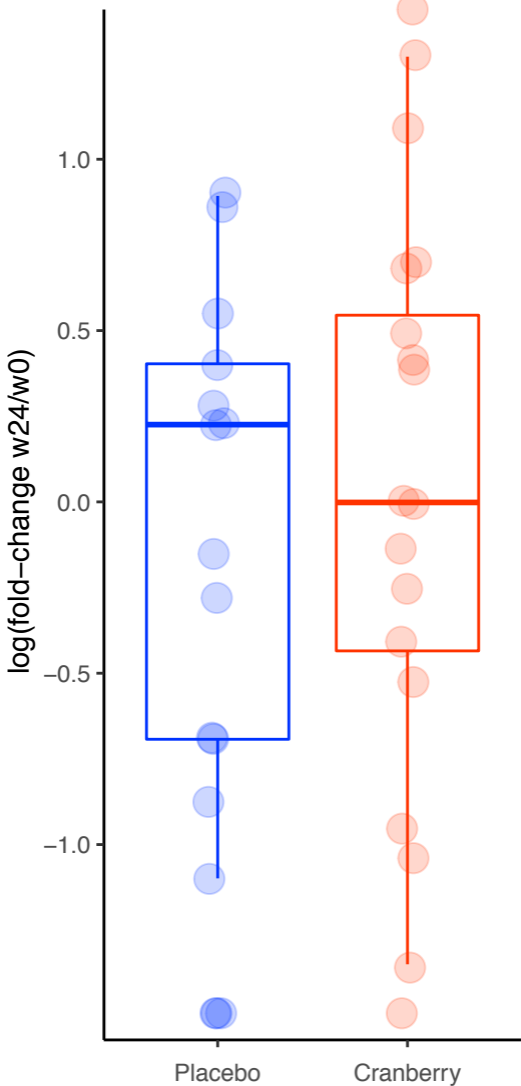

Supplement: Supplementary file 12 — Additional file 12: Figure S5. The relative abundance of OTU66, the OTU most closely related to Flavonifractor plautii, did not change significantly after cranberry beverage consumption (p = 0.43). [file 12866_2021_2106_MOESM12_ESM.pdf]

# Figure S6

A.

Allele 183G 183A 183T

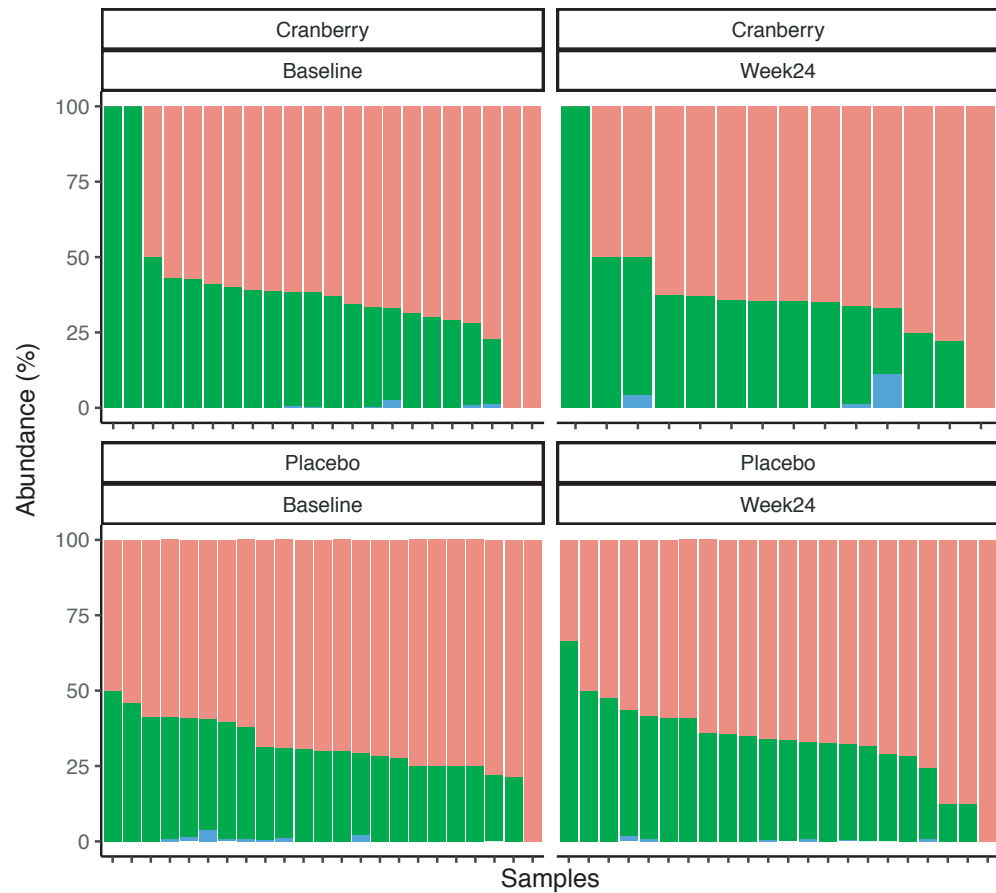

B.

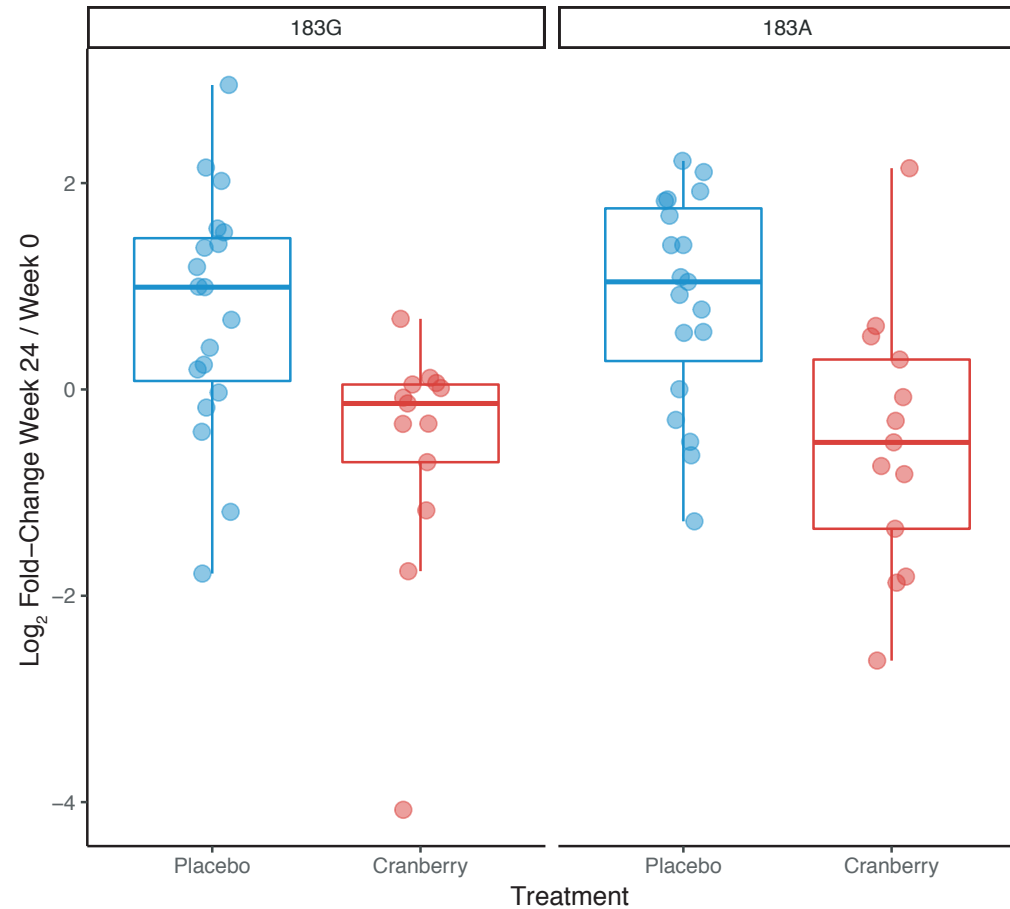

Supplement: Supplementary file 13 — Additional file 13: Figure S6. OTU41 consists of two major oligotypes, 183G and 183A. a) 183G was found in approximately two-thirds of the sequence assigned to OTU41, while 183A was found in approximately one-third, with very minor amounts of 183 T. Both 183G and 183A alleles were represented in women from both study arms at weeks 0 and 24. b) Both major oligotypes of OTU41 behave consistently with the overall behavior of OTU41, trending upwards in placebo and downwards in cranberry consumption. [file 12866_2021_2106_MOESM13_ESM.pdf]

Figure S7

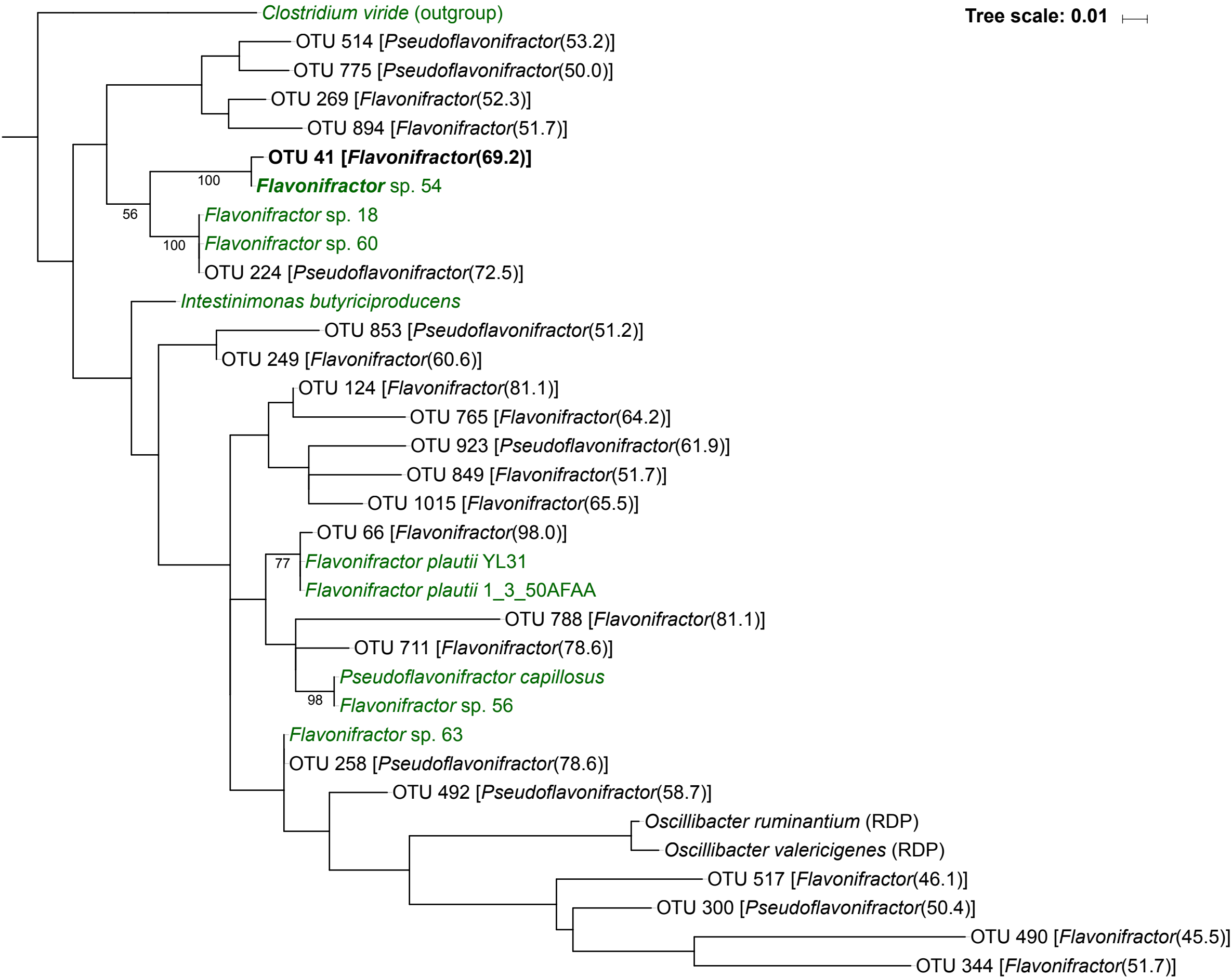

Supplement: Supplementary file 14 — Additional file 14: Figure S7. 16S rRNA V4 region phylogeny containing OTUs from our study, along with sequences extracted from the reference genomes used in the comparative genomics analysis (shown in green). OTU41 and Flavonifractor sp. 54 are indicated in bold. Taxonomic assignments for OTUs are shown in brackets, with their confidence value in parentheses. These reference genomes also showed close relationships to OTUs found across our study, indicating the species they represent are also present in study participants, though their relative abundances were unchanged by cranberry or placebo consumption. [file 12866_2021_2106_MOESM14_ESM.pdf]

Figure S8

A.

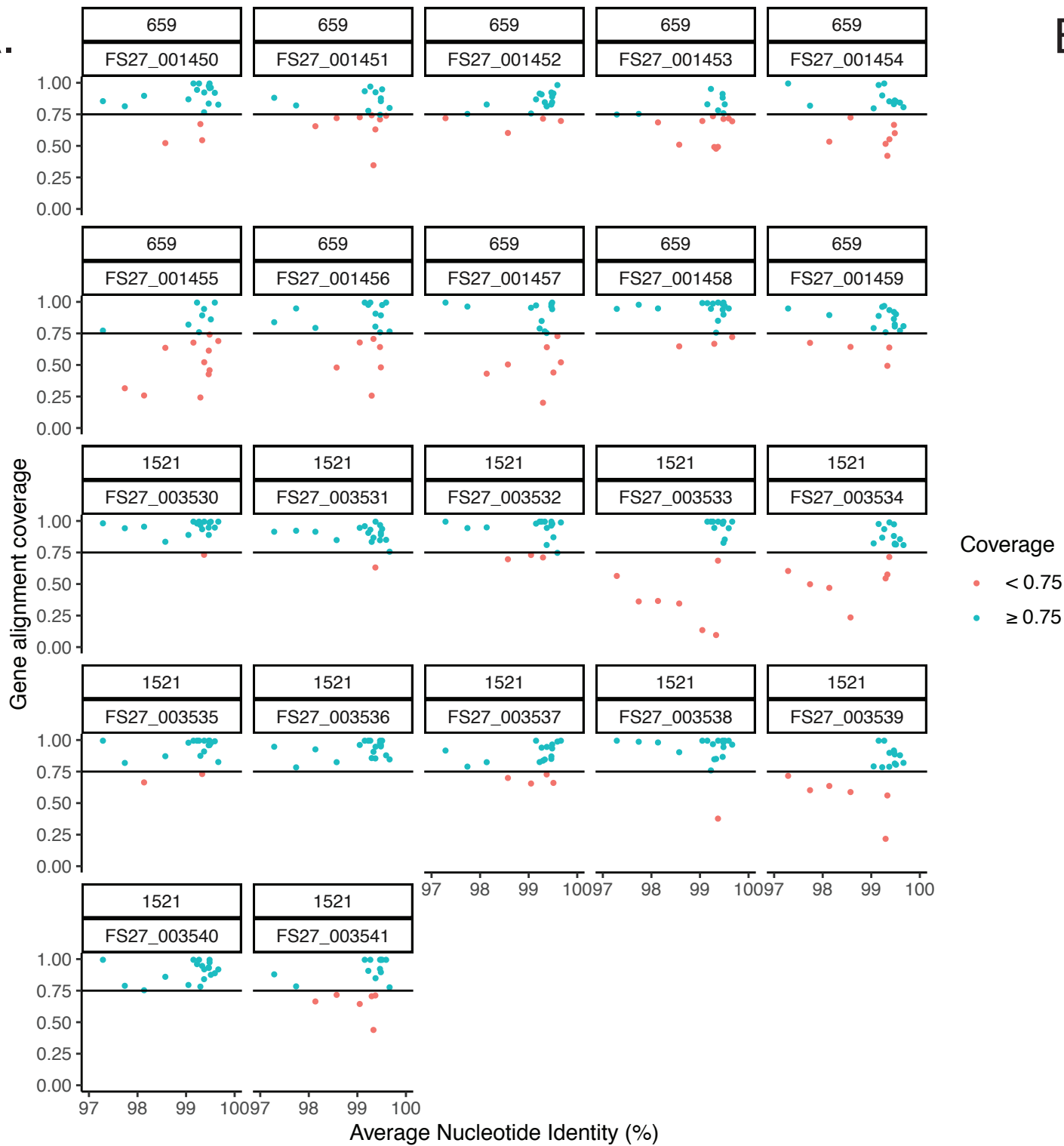

B.

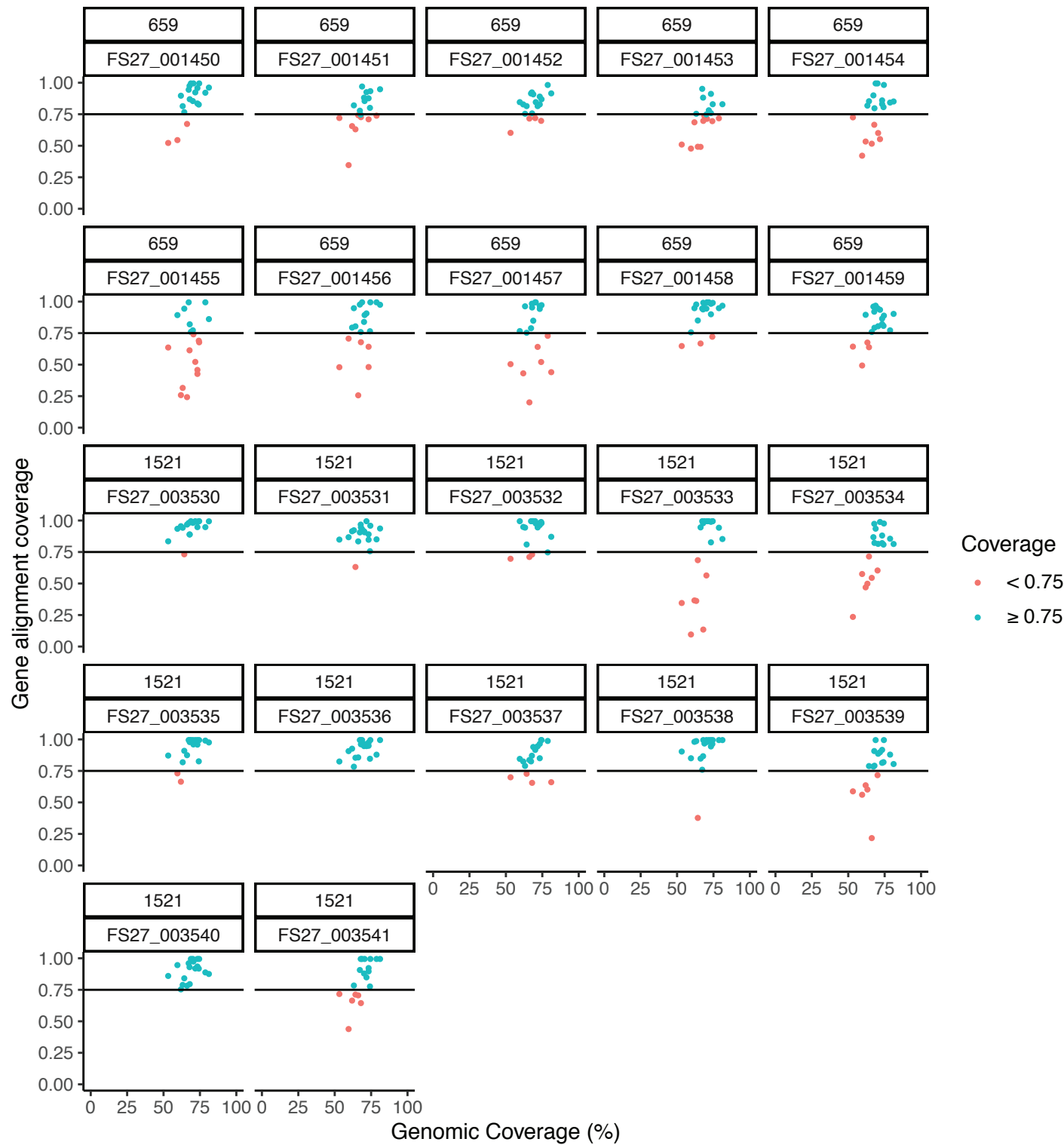

C.

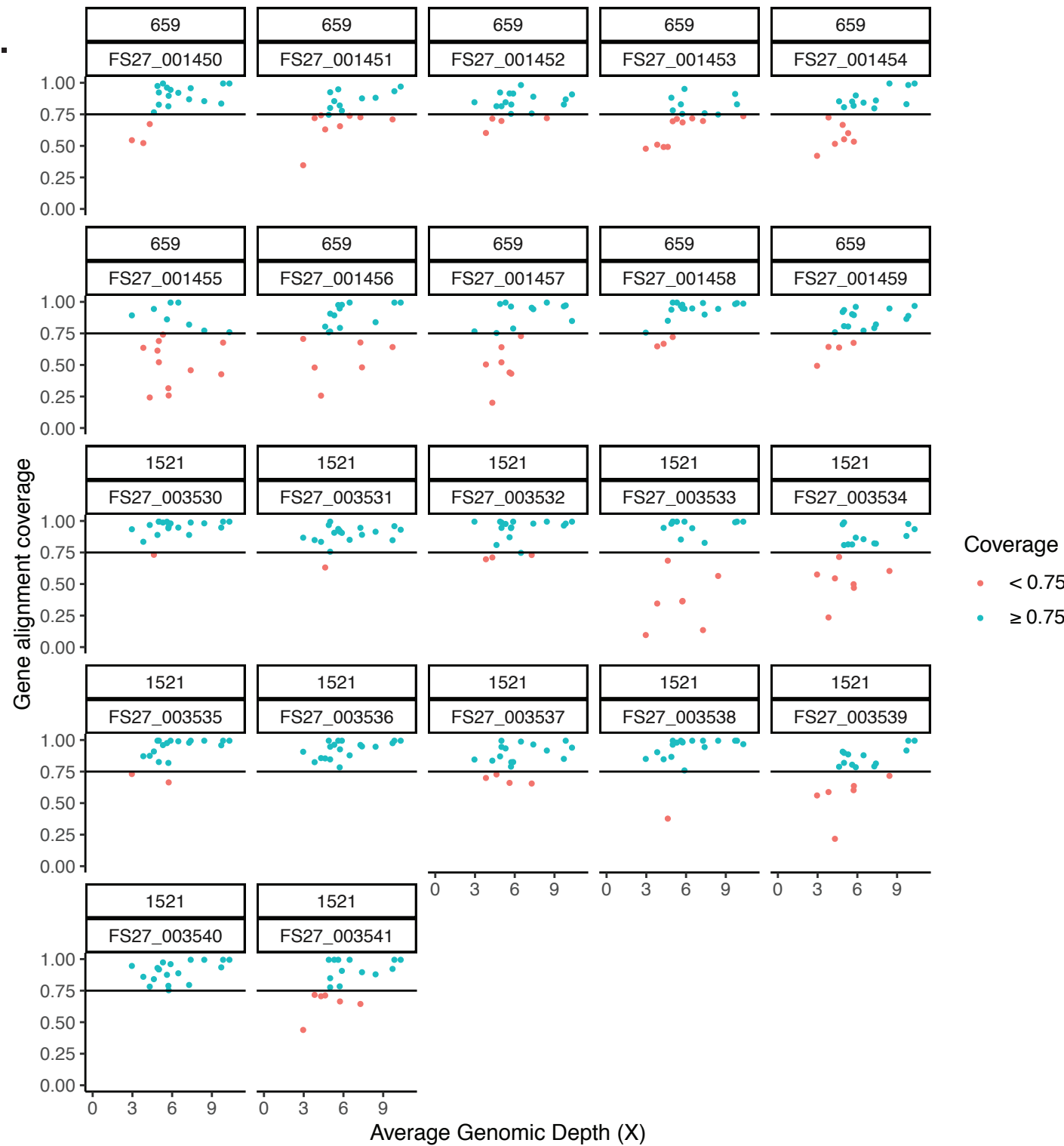

Supplement: Supplementary file 15 — Additional file 15: Figure S8. WMS alignment results of genes from two cobalamin synthesis operons in the Flavonifractor sp. 54 assembly. a) Genomic ANI to Flavonifractor sp. 54 vs. gene alignment coverage of each gene. b) Genomic breadth of coverage (in % of genome) vs. gene alignment coverage. c) Average genomic depth of sequencing vs. gene alignment coverage. These results suggest that our choice of a 75% gene coverage threshold for gene presence was conservative as OTU41-containing samples with low OTU41 abundance and/or sequencing depth tended to not meet this threshold despite modest evidence for these genes being present. [file 12866_2021_2106_MOESM15_ESM.pdf]

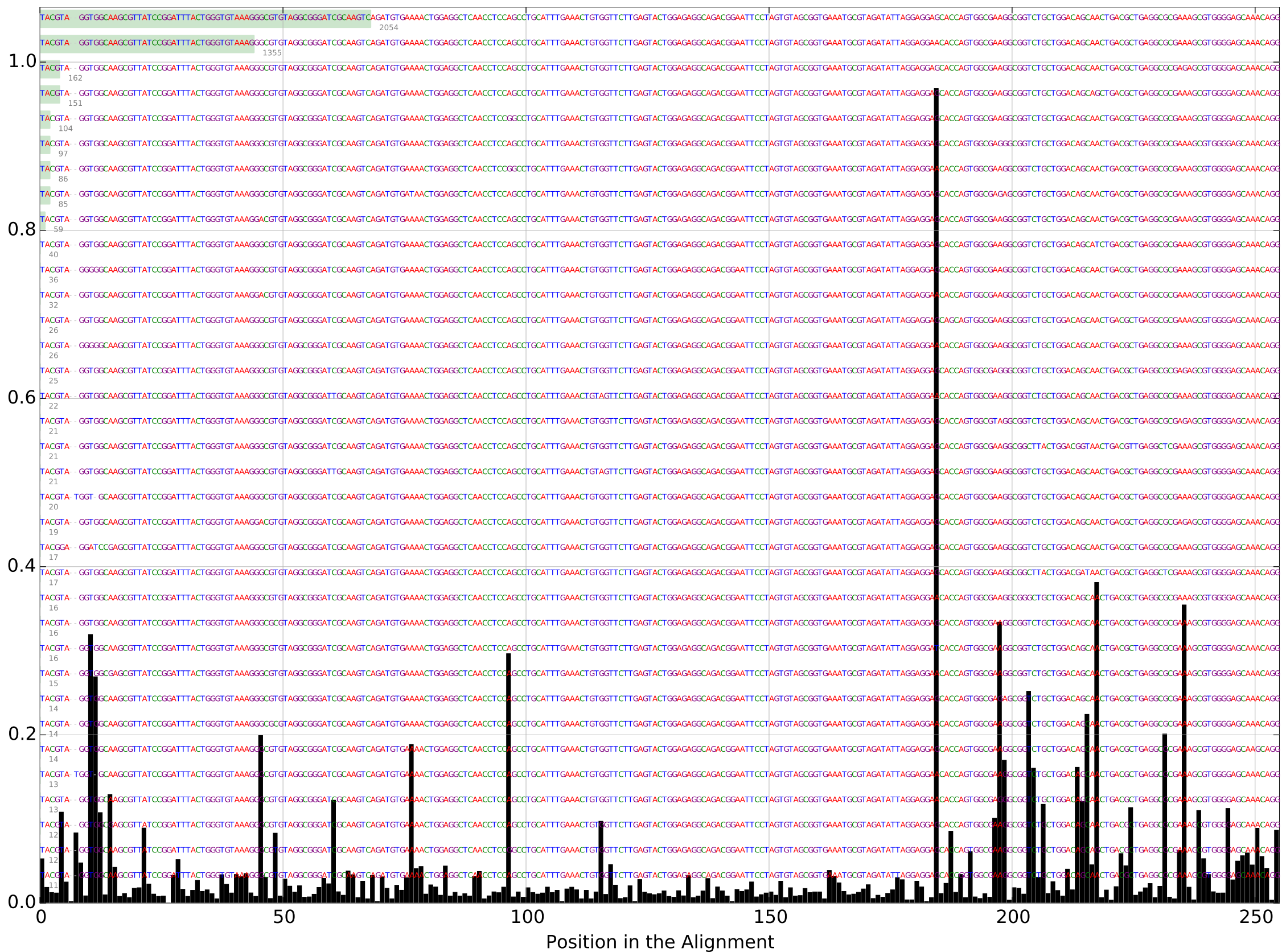

Supplement: Supplementary file 16 — Additional file 16: Figure S9. Oligotyping entropy profile for OTU41. X axis is the position in the alignment to OTU41 consensus sequence. Y axis is the Shannon Entropy of each position in the sequences that were clustered into OTU41. Position 183 was the only position in the OTU41 alignment that was above an entropy of 0.4. The background highlights the major oligotypes based on the top 6 most entropic positions, which represent the G and A alleles at position 183. [file 12866_2021_2106_MOESM16_ESM.pdf]
