# Supplementary material for: Limited effects of long-term daily cranberry consumption on the gut microbiome in a placebo-controlled study of women with recurrent urinary tract infections
Source: BMC Microbiol. 2021 Feb 18;21:53. doi: 10.1186/s12866-021-02106-4 (PMC7890861; doi:10.1186/s12866-021-02106-4)
Supplement: Supplementary file 17 — Additional file 17: Table S1. 16S rRNA sequences included in phylogenetic analysis. Table S2. Whole genomes included in comparative analysis. [file 12866_2021_2106_MOESM17_ESM.docx]

**Table S1. 16S rRNA sequences included in phylogenetic analysis**

| **Strain** | **GenBank Identifier** | **Reference/Source** |
| --- | --- | --- |
| *Lachnospiraceae* ING2-E1K | KX826959.1 | Leibniz Institute for Agricultural Engineering and Bioeconomy (ATB) |
| *Lachnospiraceae* ING2E5F | KX826958.1 | Leibniz Institute for Agricultural Engineering and Bioeconomy (ATB) |
| *Flintibacter butyricus* BLS21 | KF447772.1 | (149) |
| *Flavonifractor* sp. 54 | GCA_900066825.1 | (51) |
| *Pseudoflavonifractor* sp. 2.1.1 | JX273469 | Wageningen University |
| *Pseudoflavonifractor* sp. 766 | HE974967 | (150) |
| *Pseudoflavonifractor capillosus* ATCC 29799 | AY136666 | National Microbiology Laboratory, Health Canada |
| *Flavonifractor plautii* NML 00A095 | AY730664 | Canadian Science Centre for Human and Animal Health |
| *Flavonifractor plautii* 3 | GU968163 | (151) |
| *Flavonifractor* OTU 517 | **-** | This study |
| *Flavonifractor* OTU 344 | **-** | This study |
| *Flavonifractor* OTU 490 | **-** | This study |
| *Flavonifractor* OTU 41 | **-** | This study |
| *Flavonifractor* OTU 894 | **-** | This study |
| *Flavonifractor* OTU 269 | **-** | This study |
| *Flavonifractor* OTU 249 | **-** | This study |
| *Flavonifractor* OTU 765 | **-** | This study |
| *Flavonifractor* OTU 124 | **-** | This study |
| *Flavonifractor* OTU 788 | **-** | This study |
| *Flavonifractor* OTU 849 | **-** | This study |
| *Flavonifractor* OTU 1015 | **-** | This study |
| *Flavonifractor* OTU 711 | **-** | This study |
| *Flavonifractor* OTU 66 | **-** | This study |

**Table S2. Whole genomes included in comparative analysis.**

| **Genome** | **GenBank accession** | **Reference/Source** |
| --- | --- | --- |
| *Flavonifractor* sp. 54 (isolate 2789STDY5834931) | GCA_900066825.1 | (51) |
| *Flavonifractor* sp. 18 (isolate 2789STDY5834895) | GCA_900066645.1 | (51) |
| *Flavonifractor sp.* 56 (isolate 2789STDY5834933) | GCA_900066835.1 | (51) |
| *Flavonifractor* sp. 60 (isolate 2789STDY5834937) | GCA_900066875.1 | (51) |
| *Flavonifractor* sp. 63 (isolate 2789STDY5834940) | GCA_900066895.1 | (51) |
| *Flavonifractor plautii* strain YL31 | GCF_001688625.2 | University of Bern |
| *Flavonifractor plautii* 1_3_50AFAA (formerly named Clostridium orbiscinens 1_3_50AFAA) | GCF_000760655.1 | Broad Institute (152) |
| *Pseudoflavonifractor capillosus* ATCC 29799 | GCF_000169255.2 | Washington University Genome Sequencing Center |
| *Intestinimonas butyriciproducens* strain AF211 | GCF_001454945.1 | Wageningen University |
| *Clostridium viride* DSM 6836 (outgroup) | GCF_000620945.1 | DOE Joint Genome Institute |
